# IntAct-U-ExM enables super-resolution imaging of isoform-specific actin networks across species

**DOI:** 10.1371/journal.pbio.3003832

**Published:** 2026-06-12

**Authors:** Anubhav Dhar, Sucheta Dey, Sanjana Mullick, Nishant Kumar Suman, Maxime C. van Zwam, Nishaant Kumar Palani Balaji, Angana Ghosh, Deepak Nair, Koen van den Dries, Sudarshan Gadadhar, Saravanan Palani

**Affiliations:** 1 Department of Biochemistry, Division of Biological Sciences, Indian Institute of Science, Bengaluru, Karnataka, India; 2 Institute for Stem Cell Science and Regenerative Medicine (BRIC-inStem), Bengaluru, Karnataka, India; 3 Regional Centre for Biotechnology (RCB), Faridabad, Haryana, India; 4 Centre for Neuroscience, Division of Biological Sciences, Indian Institute of Science, Bengaluru, Karnataka, India; 5 Department of Medical BioSciences, Radboud University Medical Center, Nijmegen, The Netherlands; University of Michigan, UNITED STATES OF AMERICA

## Abstract

Expansion microscopy (ExM) has revolutionized super-resolution imaging in cell biology due to its simple and inexpensive workflow. The use of ExM has revealed several novel insights into the nanoscale architectures of cellular protein complexes, especially the microtubule cytoskeleton in model and non-model systems. Despite tremendous progress in expansion microscopy protocols that preserve cellular ultrastructure (U-ExM), compatible probes for imaging actin isoforms with U-ExM are still lacking and have hindered the study of diverse actin isoforms and networks across model systems. Here, we use IntAct, an internally tagged actin that incorporates into cellular actin networks, to develop and optimize U-ExM for diverse actin structures in yeast, mammalian cells, and primary neurons. Using ALFA-tagged IntAct variants, we achieve robust visualization of actin patches, cables, and rings in yeast, as well as diverse actin architectures including the cortex, stress fibers, filopodia, and lamellipodia in mammalian cells at improved resolution. In primary hippocampal neurons, IntAct efficiently labels actin throughout the soma and neuronal projections, revealing strong enrichment at dendritic spines and synaptic boutons. Notably, we observe a periodic organization of F-actin along axons consistent with the membrane-associated periodic cytoskeleton, thereby resolving the periodic, sub-diffraction actin ring organization. We also detect transient nuclear actin filaments using IntAct-U-ExM underscoring the advantages offered by our approach to image understudied actin structures. Overall, we demonstrate the effectiveness of IntAct-U-ExM for performing super-resolution imaging of various actin structures in an isoform-specific manner and highlight the potential of IntAct to study the nanoscale organization of diverse actin cytoskeletal networks across species.

## Abstract 2

A previous study in PLOS Biology developed IntAct, a tool to study isoform-specific actin localization, dynamics and molecular interactions across species. In this Update Article, IntAct is coupled with ultrastructure expansion microscopy to enable the super-resolution imaging of various actin structures in yeast and mammalian cells.

## Introduction

Super-resolution fluorescence microscopy techniques have revolutionized the field of cell biology over the last two decades [1,2]. The need to visualize biomolecules at ever-increasing resolution below the diffraction limit has led to the development of advanced techniques like STED [3,4], SIM [5–7], STORM [8], PALM [9,10], DNA-PAINT [11–13], MINFLUX [14,15]. A recent addition to this list, expansion microscopy (ExM) began as a qualitative method for imaging biological samples in the Boyden lab in 2015 [16], but has evolved rapidly in the last decade to offer spatial information comparable to super-resolution microscopy. It is a first-of-its kind technique which uses physical expansion of the biological specimen instead of advanced optics and computation to circumvent the diffraction limit [17,18]. The Guichard and Hamel lab at the University of Geneva modified this protocol in 2019, where they enabled preservation of the ultrastructure of cellular organelles during the expansion procedure that enabled visualization of diverse cellular ultrastructures at nanometer resolution (U-ExM) [19], which has since become one of the most routinely used protocols for ExM. Over the years, several iterations of the original ExM protocol have significantly made the workflow more user-friendly, accessible to any molecular biology laboratory, improved reproducibility, and extended applicability for various cell types and species [20–24]. Innovations in hydrogel chemistry underlying expansion has enabled advancement in expansion factors and combination with other super-resolution modalities have greatly increased the achievable molecular resolution [22,23,25–29].

ExM involves embedding and crosslinking the biological specimen in a swellable hydrogel that physically expands in volume by absorbing water. The sample is denatured after embedding in the gel to allow isotropic expansion of the biological specimen with minimal changes to the cellular architecture. The biomolecule-of-interest can be visualized by staining the hydrogel with antibodies or other stains pre/post-expansion of the sample and imaging the sample with any conventional widefield or confocal microscopes. Denaturation and post-expansion labeling can make epitopes more accessible, allowing many antibodies, especially those that recognize denatured proteins used in western blots to work well in ExM [30]. Thus, there exists a net-positive trade-off where some antibodies or staining reagents may lose binding capability due to a denatured epitope, but overall, the process tends to improve staining compatibility. These advances have allowed visualizing various cellular structures including the cytoskeletal filaments of tubulin across species [31,32]. Despite this progress, visualizing actin via ExM has remained challenging due to a lack of probes.

Actin cytoskeleton plays diverse roles in countless cellular processes and the nanoscale organization of actin and actin-binding proteins within different actin networks is an active area of research [33–41]. Recent studies have developed modified phalloidin conjugates that enable visualization of F-actin structures in expanded samples [42–44]. However, they introduce high linkage errors, show inefficient F-actin binding due to the bulky nature of the antibodies used, and are incompatible with heat denaturation and post-expansion labeling in the widely used U-ExM protocol. Additionally, they do not provide isoform-specific information on actin and their use in ExM studies has been severely limited post-development. Apart from modified phalloidin probes, anti-actin antibodies can be used to label actin post-expansion [45,46], but they suffer from high linkage error, cytoplasmic background, poor labeling post-denaturation of epitope, and low-labeling densities insufficient for ExM [42]. In addition, anti-actin antibodies require careful optimization of fixation and labeling conditions [45–47], and unlike tubulin, pan anti-actin antibodies that can reliably label actin post-expansion across species have not yet been reported [31], making them a less ideal candidate. Thus, there is an urgent need to develop universal and versatile probes for actin compatible with U-ExM.

In this study, we have developed and optimized a protocol for performing U-ExM of actin isoforms in yeast and mammalian cells. Previously, we have reported a permissive site for epitope tag insertion within the actin protein (T229/A230), called “IntAct”, which shows isoform-specific incorporation into native actin filaments across species [47]. Here, by expressing IntAct actin variants with an ALFA tag [48], we achieve clear post-expansion labeling of specific actin isoforms in mammalian and yeast cells using the nanobody against the ALFA tag [48] (NbALFA) with a good signal-to-noise ratio. Importantly, this approach extends to primary neurons, enabling direct visualization of actin organization within complex cellular architectures. Our IntAct strategy enables visualization of actin network organization below the diffraction limit in cells from diverse model systems in an isoform-specific manner, opening vast possibilities towards understanding the nanoscale architecture and functions of actin filaments across various network types.

## Results

### IntAct enables ultrastructure expansion microscopy of actin networks in yeast cells

**Fig 1:**
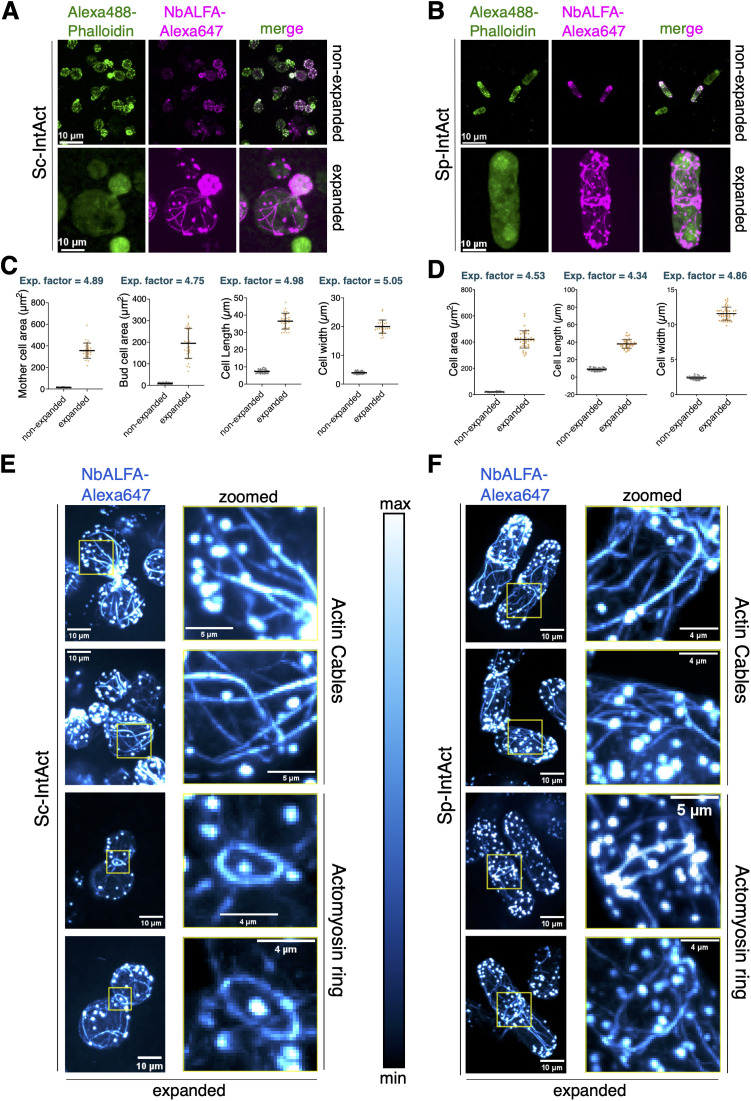
IntAct-U-ExM enables visualization of actin patches, cables, and rings in budding and fission yeasts. **(A)** Representative maximum intensity projected images of non-expanded and expanded *Saccharomyces cerevisiae* cells expressing Sc-IntAct stained as indicated. **(B)** Representative maximum intensity projected images of non-expanded and expanded *Schizosaccharomyces pombe* cells expressing Sp-IntAct stained as indicated. **(C)** Plots representing measurements of *S. cerevisiae* mother cell area, bud cell area, cell length (mother tip to bud tip), mother cell width in non-expanded and expanded samples and calculated expansion factors (*n* ≥ 28 cells). **(D)** Plots representing measurements of *S. pombe* cell area, cell length, cell width (at cell equator) in non-expanded and expanded samples and calculated expansion factors (*n* ≥ 29 cells). **(E)** Representative maximum intensity projected images of expanded *S. cerevisiae* cells stained with NbALFA-Alexa647 showing actin patches, cables, and rings. **(F)** Representative maximum intensity projected images of expanded *S. pombe* cells stained with NbALFA-Alexa647 showing actin patches, cables, and rings. (LUT display range is indicated as a vertical bar in the figure). The numerical data underlying this figure can be found in S1 Data.

The yeast actin cytoskeleton consists of three major actin structures: (1) Actin cables—bundles of linear actin filaments nucleated by formin proteins [49,50], (2) Actin patches—branched actin networks at endocytic sites nucleated by the Arp2/3 complex [51,52], (3) Actin rings—bundled actin filaments at the mother-bud neck nucleated by formins [53,54]. Previously, we have shown that IntAct can incorporate in all these three actin structures when expressed in budding and fission yeast [47]. We reasoned that IntAct’s efficient incorporation into native actin structures, minimal disruption to filament dynamics, and the known stability and versatility of the small internal ALFA tag to perform various applications could allow reliable staining with NbALFA [48,55], enabling super-resolution analysis of actin isoforms using ExM. To test this, we used *Saccharomyces cerevisiae* and *Schizosaccharomyces pombe* strains that expressed their native IntAct proteins from an exogenous plasmid copy and performed U-ExM using a recently optimized protocol for yeast [56,57]. Imaging *S. cerevisiae (S.c.)* and *S. pombe* (*S.p.*) cells revealed staining of actin structures with NbALFA-Alexa647 in both the non-expanded and expanded cells. Interestingly, detection of actin cables was significantly better in expanded samples, suggesting increased accessibility of the ALFA tag for NbALFA binding post-denaturation and expansion (Fig 1A and 1B). In contrast, Alexa488-Phalloidin could stain actin structures only in non-expanded cells and no clear staining was observed in expanded cells (Fig 1A and 1B), consistent with the known incompatibility of fluorescent dye-conjugated phalloidin with ExM [42,43]. We measured cell area and dimensions and observed an average expansion factor of 4.92 for *S. cerevisiae* (Fig 1C) and 4.58 for *S. pombe* (Fig 1D), as compared to non-expanded cells.

Despite successful staining of actin structures post-expansion, we observed that actin cables in both yeasts were not well preserved and showed discontinuous staining along their length (S1A and S1B Fig). To improve this, we compared NbALFA staining in cells fixed with 4% formaldehyde (FA) or a mix of 4% FA + 0.1% glutaraldehyde (GA), which has been shown to improve preservation of native microtubules during U-ExM previously [45]. We observed that cells fixed with 4% FA + 0.1% GA showed significantly better preservation and uniform staining of actin cables (S1A and S1B Fig). With this optimized protocol, we successfully and consistently imaged actin patches, actin cables, and actomyosin rings in the yeasts *S. cerevisiae* (Fig 1E) and *S. pombe* (Fig 1F). Notably, we were able to image actin cables, which are low-actin dense structures and difficult to visualize with traditional staining, with unprecedented detail and resolution, establishing IntAct-U-ExM as a powerful new way to study actin cable architecture across species. The actin cables post-expansion showed an average scaled FWHM of 74.89 ± 12.15 nm for *S.c.* (S1C Fig) and 66.17 ± 9.08 nm for *S.p.* (S1D Fig), which is close to a previous measurement of 60 nm from an EM study in *S. pombe* [58]. The cytokinetic actin rings showed a diameter of 4.15 µm ± 0.56 µm for *S.c.* (S1E Fig) and 10.80 µm ± 0.61 µm for *S.p.* (S1E Fig), corroborating a ~4.0 to 4.5 expansion factor. Overall, the above results demonstrate: (i) the application of IntAct to enable U-ExM of actin structures in yeast and its potential for quantitative super-resolution imaging of actin networks across the fungal kingdom, (ii) establishes the ALFA tag-NbALFA pair as an excellent way to image target proteins/structures with U-ExM, adding to the list of validated epitope tags for use in ExM.

### IntAct enables isoform-specific expansion microscopy of actin networks in cultured mammalian cells

**Fig 2:**
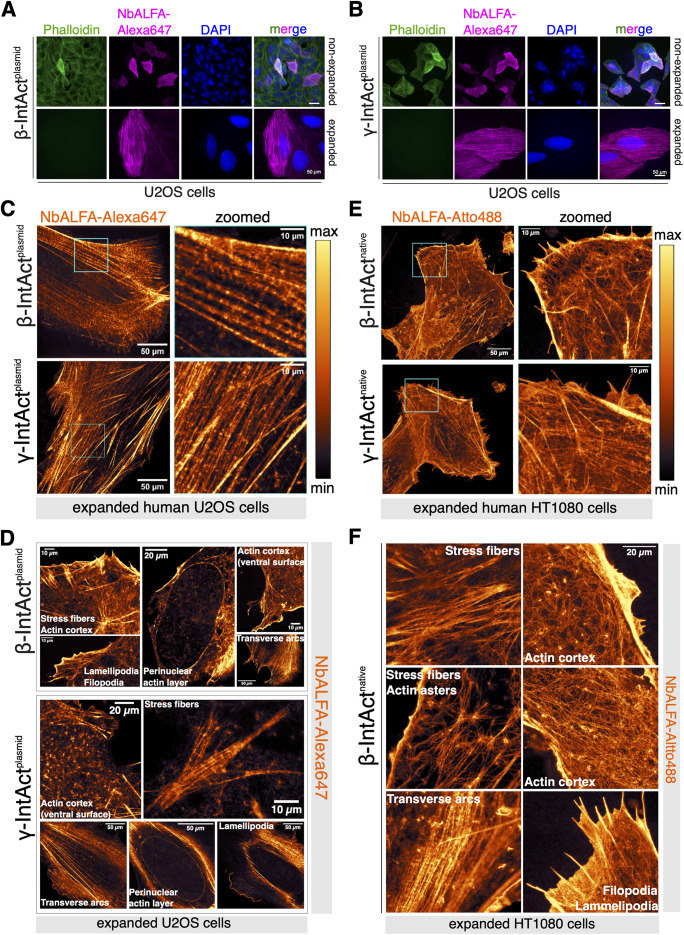
IntAct-U-ExM can be used to study mammalian actin networks in an isoform-specific manner. **(A)** Representative maximum intensity projected images of non-expanded and expanded human U2OS cells expressing β-IntAct stained as indicated. **(B)** Representative maximum intensity projected images of non-expanded and expanded human U2OS cells expressing γ-IntAct stained as indicated. **(C)** Representative maximum intensity projected images of expanded human U2OS cells expressing either β- or γ-IntAct from a transfected plasmid copy stained with NbALFA-Alexa647 showing various actin structures. **(D)** Representative single plane or maximum intensity projected images of expanded human U2OS cells expressing either β- or γ-IntAct stained with NbALFA-Alexa647 showing diversity of actin filament networks as indicated. **(E)** Representative maximum intensity projected Airyscan images of expanded human HT1080 cells expressing β-IntAct or γ-IntAct from their respective endogenous genomic loci stained with NbALFA-Atto488 showing various actin structures. **(F)** Representative single plane or maximum intensity projected Airyscan images of expanded human HT1080 cells expressing β-IntAct from its endogenous genomic loci, stained with NbALFA-Atto488 showing diversity of actin filament networks as indicated. (LUT display range is indicated as a vertical bar in the figure).

The success of IntAct in enabling U-ExM of yeast prompted us to test its applicability in cultured mammalian cells which harbor 6 isoforms of actin that express in different tissue types [33,59]. We used human osteosarcoma U2OS cells and specifically expressed the human non-muscle beta (β)- or gamma (γ)-IntAct isoforms in these cells from a transiently transfected exogenous plasmid. Transfected cells were prepared for U-ExM using a previously described protocol for human cells [19] and imaged with either an epifluorescence or a spinning-disk confocal microscope. We observed clear staining of actin filaments in expanded U2OS cells expressing either β-IntAct and γ-IntAct (Fig 2A and 2B). Consistent with previous studies [43] and our experiments with yeast, phalloidin only stained actin structures in non-expanded U2OS cells and did not stain actin structures post-expansion in U2OS cells (Fig 2A and 2B). The cells showed an average expansion factor of 3.29 and 3.84 for β- and γ-IntAct expressing U2OS cells as measured by the increase in nucleus area (S2A Fig). The relatively lower expansion factor observed for nuclei may be due to the fact that all cellular compartments don’t expand with the same factor as observed previously and changes in cross-linker composition can help mitigate such effects [60]. We consistently observed robust staining of β- and γ-IntAct filaments with clearly improved resolution in various actin networks such as stress fibers [61], actin cortex [62], lamellipodium [63], filopodia [64], etc. throughout the volume of expanded U2OS cells (Figs 2C, 2D, and S2B). To validate these results in another mammalian model, we expressed and imaged human beta (β)- or gamma (γ)-IntAct isoforms in non-expanded and expanded mouse neuroblastoma, Neuro-2a (N2a) cells, and observed clear staining of various actin structures post-expansion (S3A, S3B, and S3D Fig). N2a cells also displayed isotropic expansion with the nucleus area increasing by an average expansion factor of 3.88 and 3.24 for β- and γ-IntAct expressing N2a cells (S3C Fig). These results highlight the significant advantage provided by IntAct-U-ExM to study mammalian actin networks at high 3D-molecular resolution and highlight the strong potential for future studies of actin and its binding proteins with multiplexed imaging [65].

Interestingly, we detected both β- and γ-IntAct signal appearing as filaments in the nucleus of few of the U2OS cells (S2C Fig) and as a continuous layer surrounding the nucleus in expanded U2OS cells (Figs 2D and S2C). Actin filaments inside the nucleus have been observed previously with the use of nuclear-targeted actin chromobody (nAC) [66] and remain challenging to visualize by other conventional labeling approaches [67]. Our results, thus, demonstrate a plausible alternate way to study these nuclear actin filaments with isoform specificity [47]. These observations demonstrate the potential of IntAct-U-ExM in elucidating lesser-studied actin filament populations in diverse cellular compartments, promising to reveal new functional aspects of actin.

Next, to assess the performance of IntAct-U-ExM when expressed at endogenous levels in mammalian cells, we used human fibrosarcoma HT1080 cells with homozygous knock-in of β-IntAct or hemizygous knock-in of γ-IntAct at their genomic loci [47]. Our results revealed clear staining of actin networks in HT1080 cells with a good signal-to-noise ratio post-expansion (Fig 2E). The cells showed an average expansion factor of 4.25, as measured by the increase in nucleus area (S2D Fig). Using Airyscan super-resolution microscopy, we consistently detected diverse actin structures such as filopodia, lamellipodia, cortical actin filaments, transverse arcs, and ventral stress fibers at very high detail (Fig 2F), indicating that IntAct-U-ExM is a reliable and robust strategy to visualize mammalian actin filaments networks at native expression levels.

### IntAct reveals nanoscale F-actin organization in primary neurons

**Fig 3:**
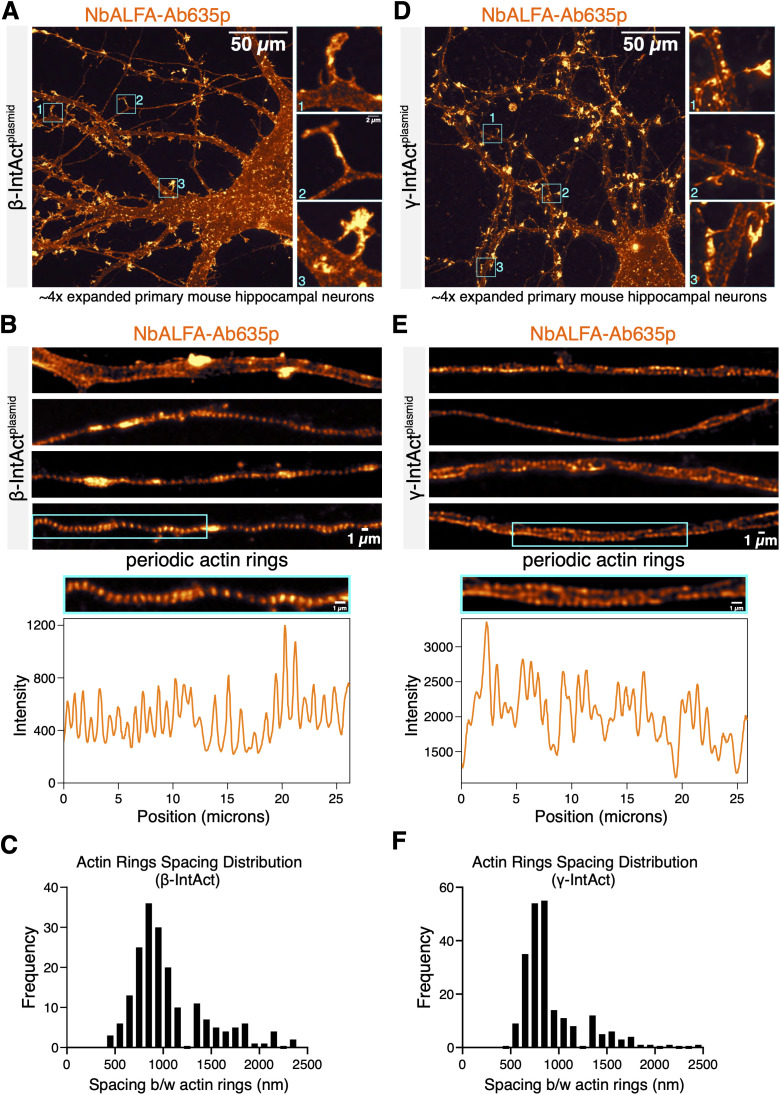
IntAct-U-ExM reveals nanoscale F-actin organization in primary mouse neurons. **(A)** Representative image of expanded primary mouse hippocampal neurons (div14) expressing β-IntAct and stained with NbALFA-Ab635p. **(B)** Representative images and line scan intensity plot depicting periodic β-IntAct organization along axons in expanded primary mouse hippocampal neurons (div14). **(C)** Frequency distribution of post-expansion spacing between periodic actin rings observed in expanded primary mouse hippocampal neurons (div14) expressing β-IntAct (*n* = 189 ring spacings). **(D)** Representative image of expanded primary mouse hippocampal neurons (div14) expressing γ-IntAct and stained with NbALFA-Ab635p. **(E)** Representative images and line scan intensity plot depicting periodic γ-IntAct organization along axons in expanded primary mouse hippocampal neurons (div14). **(F)** Frequency distribution of post-expansion spacing between periodic actin rings observed in expanded primary mouse hippocampal neurons (div14) expressing γ-IntAct (*n* = 220 ring spacings). The numerical data underlying this figure can be found in S1 Data.

To validate the utility of IntAct-U-ExM beyond cultured cell lines and genetically pliable systems, we tested its use in primary mouse hippocampal neurons. We transfected β-IntAct and γ-IntAct containing expression plasmids in isolated primary hippocampal neurons. Neurons were allowed to mature for 14 days post-isolation, a timeframe critical for the establishment of complex dendritic arborizations, axonal polarization, and robust synaptogenesis, and subsequently fixed and prepared for U-ExM. We observed good incorporation and staining of IntAct throughout the neuronal cell body and projections (dendrites and axons) in both non-expanded and expanded cells (Figs S3E, 3A, and 3C). F-actin accumulation was robustly observed at dendritic spines, where localized actin dynamics are essential for spine morphogenesis and synaptic plasticity, and at synaptic boutons along axons, where actin networks regulate synaptic vesicle pooling and release (Fig 3A and 3C), as previously shown.

Interestingly, periodic staining of both β-IntAct and γ-IntAct was observed along most of the axonal projections (Fig 3B and 3D), reminiscent of the Membrane-associated Periodic Cytoskeleton (MPS) present in axons [68, 69]. The MPS contains a highly ordered, periodic ring-like arrangement of F-actin crosslinked with the actin-binding protein spectrin, and the actin rings are known to have a periodic spacing of ~190 nm [68]. Because this spacing falls well below the diffraction limit of light, it is notoriously difficult to resolve without specialized super-resolution techniques or probes with low linkage error. To confirm whether the observed periodic staining indeed represents actin rings, we analyzed fluorescence intensity line scans to measure the distance between each pair of adjacent rings in our expanded samples. Since we consistently observed expansion factors between 4 and 5 throughout the study, the expected post-expansion distances between the actin rings should lie between 760 nm (4*190 nm) and 950 nm (5*190 nm). Our analysis revealed a median ring spacing of 975 nm for β-IntAct and 866 nm for γ-IntAct. The frequency distribution analysis revealed that the most frequent ring spacing lied in the expected range of 800–900 nm for both β-IntAct and γ-IntAct (Fig 3C and 3F), confirming that periodic IntAct staining indeed represents sub-diffraction-limited neuronal actin rings (MPS). Since there is no prior work to suggest differences in ring spacing between β-actin and γ-actin, the higher median ring spacing observed for β-IntAct is likely a result of measurement scatter or heterogeneity in local expansion factors, or a combination of both. This work is thus the first demonstration of the presence of both β- and γ-actin isoforms in the periodic actin rings in neurons, and careful future evaluation and corroboration with other super-resolution techniques like STED, SMLM, SIM will be required to delineate the respective contributions of different actin isoforms in the MPS in neurons.

These results demonstrate the applicability of IntAct-U-ExM to primary cell lines and its direct utility in revealing sub-diffraction-limited actin filament organization. Furthermore, the neuronal actin rings are known to be difficult to stain and resolve with ExM as per previous attempts [41] and the successful visualization of these delicate structures by IntAct-U-ExM is an outstanding proof of its potential for dissecting the distinct, isoform-specific [47] roles of actin in complex cellular models.

### Comparison of IntAct-U-ExM and HAK-actin for super-resolution imaging of diverse actin networks

To further evaluate the performance and versatility of IntAct-U-ExM, we compared IntAct-U-ExM to another recently developed U-ExM actin probe, HAK-actin [70]. HAK-actin is a modified form of the Jasplakinolide peptide derived from marine sponges of the genus *Jaspis* [71]. HAK-actin contains a Jasplakinolide peptide conjugated to an HA-tag and is stained post-expansion by anti-HA antibodies during U-ExM. We observed that both IntAct and HAK-actin robustly label actin structures in mammalian cell lines, performing equally well in U2OS and HT1080 cells (S4A and S4B Fig). Further, both probes successfully visualized F-actin organization in primary mouse hippocampal neurons (S4C Fig). While IntAct consistently provided superior detection of periodic axonal actin rings as compared to HAK-actin post-expansion in our hands, we note that these qualitative differences could emerge from variations in experimental conditions and will require further validation. Crucially, a major difference in probe performance was observed in fungal cells. HAK-actin did not successfully stain actin networks post-expansion in *S. cerevisiae* and *S. pombe* (S4D Fig). In contrast, IntAct produced robust and reliable staining of yeast actin structures following expansion, and to our knowledge, remains the only probe currently capable of effectively labeling yeast actin networks in ExM.

Overall, IntAct and HAK-actin represent highly useful, complementary tools for studying F-actin organization with ExM, each offering distinct experimental advantages.

### IntAct-U-ExM is compatible with various pharmacological signaling perturbations of the actin cytoskeleton

**Fig 4:**
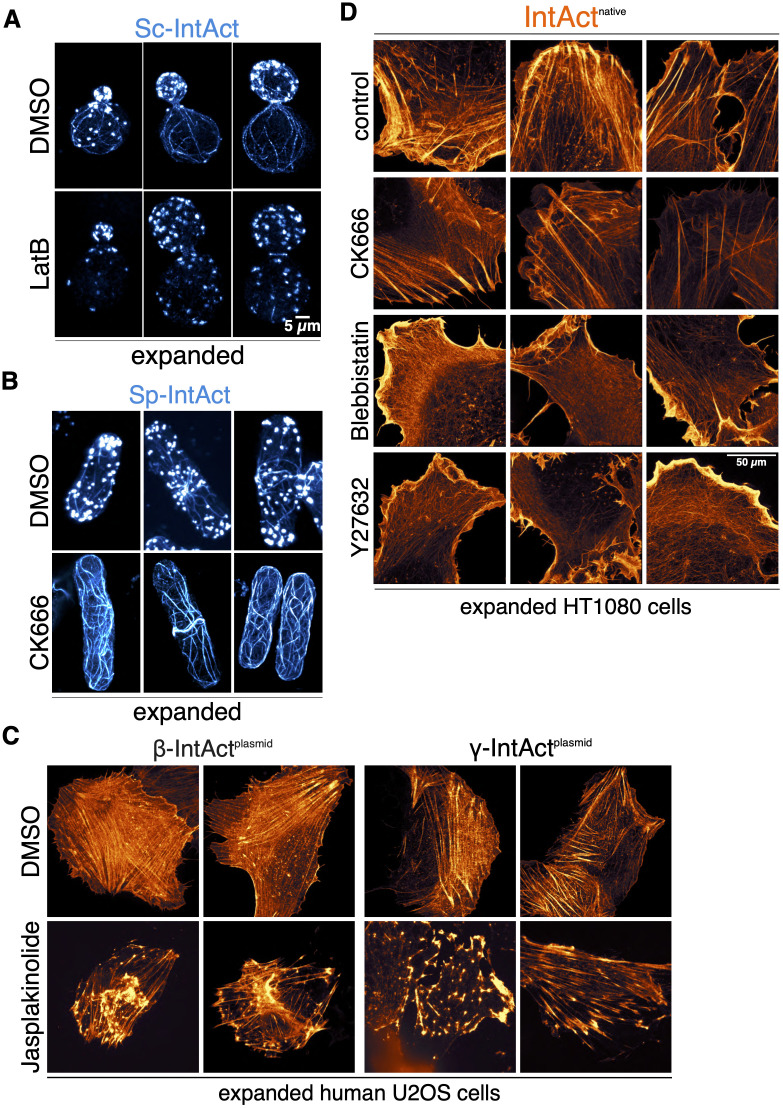
IntAct-U-ExM is compatible with a wide variety of pharmacological signaling perturbations. **(A)** Representative images of expanded and stained *Saccharomyces cerevisiae* cells expressing Sc-IntAct treated with DMSO or 50 µM Latrunculin B. **(B)** Representative images of expanded and stained *Schizosaccharomyces pombe* cells expressing Sp-IntAct treated with DMSO or 100 µM CK666. **(C)** Representative images of expanded and stained U2OS cells expressing β- or γ-IntAct treated with DMSO or 500 nM Jasplakinolide. **(D)** Representative images of expanded and stained HT1080 cells expressing β- or γ-IntAct treated with DMSO or 100 µM CK666, or 20 µM Blebbistatin, or 20 µM Y27632.

To test the compatibility of IntAct-U-ExM with experimental perturbations to the actin cytoskeleton, we evaluated its performance following treatment with several well-characterized chemical inhibitors across diverse model systems (Table 2). First, we exposed *S. cerevisiae* cells to a mild concentration of the actin-monomer sequestering drug, Latrunculin B (LatB) [72] treatment for 15 min. Following expansion, we observed a near-complete loss of actin cables, whereas actin patches remained largely intact (Fig 4A). This demonstrates that IntAct faithfully captures expected dose-sensitive responses to LatB, highlighting its compatibility with a wide range of concentration regimes rather than being limited to high concentrations that induce total F-actin depolymerization. Next, we treated *S. pombe* cells with CK666 [73], an inhibitor of the Arp2/3 complex. Upon U-ExM imaging, we observed the expected loss of branched F-actin-containing patches, accompanied by an increase in the abundance of formin-nucleated actin cables (Fig 4B). This observation is consistent with previous studies demonstrating the channeling of free G-actin monomers into linear actin networks following the inhibition of branched actin assembly [74].

**Table 2:** Details of chemical inhibitors used in this study.

Chemical	Manufacturer (Catalog No.)	Concentration used	Treatment duration	Organism
Latrunculin B	Sigma Aldrich (428020)	50 µM	15 min	*S. cerevisiae*
CK666	Sigma Aldrich (SML0006)	100 µM	15 min	*S. pombe*
Jasplakinolide	Invitrogen (J7473)	500 nM	30 min	U2OS cells
CK666	Sigma Aldrich (SML0006)	100 µM	30 min	HT1080 cells
Blebbistatin	Cayman chemicals (13013)	20 µM	60 min	HT1080 cells
Y27632	Selleckchem (S1049)	20 µM	60 min	HT1080 cells

To extend these findings to mammalian systems, we treated U2OS cells expressing exogenous IntAct with jasplakinolide [71,75], an F-actin stabilizing peptide. Post-expansion imaging revealed decreased cell spreading and the formation of F-actin aggregates (Fig 4C), aligning with established responses to this drug [76,77]. Finally, we subjected HT1080 cells endogenously expressing IntAct to a panel of chemical inhibitors, including CK666, blebbistatin [78] (a myosin-II inhibitor), and Y27632 (a ROCK kinase inhibitor). Compared to DMSO-treated controls, cells treated with CK666 exhibited a subtle but clear reduction in cortical and lamellipodial actin at the cell periphery (Fig 4D). Furthermore, treatment with either blebbistatin or Y27632 resulted in a distinct loss of stress fibers (Fig 4D), consistent with previous literature on the inhibition of actomyosin contractility [79,80].

Taken together, these results confirm the robust compatibility of IntAct with various pharmacological perturbations to the actin cytoskeleton. This firmly establishes the applicability of IntAct-U-ExM as a reliable approach for studying nanoscale F-actin organization under a wide array of experimental and biological conditions.

## Discussion

In this study, we have developed and demonstrated the versatile application of IntAct-U-ExM as an effective method to study diverse actin-based structures with super-resolution ExM in yeast, mammalian cells, and primary neurons (S5 Fig). We successfully visualized actin patches, cables, and actomyosin rings in both budding and fission yeasts. In mammalian cell lines expressing IntAct either exogenously or natively, we reliably labeled diverse, complex networks including the actin cortex, stress fibers, lamellipodia, and filopodia. Furthermore, in primary neurons, we visualized sub-diffraction-limited periodic axonal actin rings, which have been very challenging to resolve with ExM [41], reflecting the power and high-resolution capabilities of the IntAct-U-ExM approach. Beyond cytoplasmic networks, we also detected distinct β- and γ-IntAct signals appearing as filaments within the nucleus of U2OS cells. Nuclear actin filaments remain challenging to visualize using other conventional labeling approaches. The successful detection of these filament populations highlights the efficient incorporation of IntAct even within the nucleus, while emphasizing the distinct benefits of post-expansion staining [30], which increases epitope accessibility in restricted subcellular compartments. These observations are a promising avenue to study filament populations such as those in the nucleus and associated with organelles, but warrant further experimental testing. Visualizing the nanoscale architecture of the actin cytoskeleton has previously required specialized and expensive microscopy hardware. By leveraging the physical expansion of biological specimens, our approach provides a highly dependable modality to study specific isoforms of actin [47] and their localized roles within diverse cellular compartments. Importantly, this methodology circumvents the need for complex optics, thereby making super-resolution imaging of actin networks significantly more accessible and affordable to researchers around the globe, democratizing high-resolution cytoskeletal research across diverse model systems.

While IntAct represents the first U-ExM-compatible F-actin probe, another compatible probe for visualizing F-actin with U-ExM, HAK-actin [70], was reported during the revision of our study. Both IntAct and HAK-actin represent significant advancements for the field and serve as highly complementary tools, each with distinct advantages and limitations. In our specific benchmarking, we observed that both probes perform exceptionally well in mammalian cell lines (U2OS and HT1080). In primary neurons, both probes labeled actin networks in the cell body and synapses well; however, IntAct-U-ExM consistently revealed the sub-diffraction-limited periodic MPS actin rings, while these actin rings were rarely resolved by HAK-actin. However, it is important to note that this difference may be a result of experimental conditions and MPS visualization by HAK-actin may require further experimental optimization. Nevertheless, resolving neuronal actin rings is a stringent benchmark for any actin cytoskeleton probe [41,69], and IntAct-U-ExM success in this metric is a strong indicator of its versatile applicability. Most notably, while HAK-actin failed to stain yeast actin networks post-expansion, IntAct successfully and consistently labeled these structures, establishing it as the only currently validated probe for post-expansion labeling of actin networks in fungal models.

IntAct-U-ExM offers significantly smaller linkage error due to the compact size of the nanobody (NbALFA), allows for actin isoform-specific imaging [47], and enables post-expansion labeling of epitopes, which improves probe penetration into dense actin networks. Conversely, HAK-actin offers the advantage of labeling endogenous actin without the need for exogenous expression or genomic tagging, and it benefits from signal amplification provided by secondary antibodies. IntAct labeling strategy may also benefit in divergent organisms (such as *Naegleria* and *Giardia*) where Jasplakinolide may fail to bind properly to F-actin. A detailed comparison of these two probes is given in Table 1.

**Table 1:** Comparison of key features of IntAct-U-ExM and HAK-actin.

	IntAct-U-ExM	HAK-actin
**Targeting Mechanism**	Nanobody (NbALFA) binding to genetically inserted ALFA tag	Jasplakinolide-derivative binding to F-actin
**Expression Requirement**	Yes (exogenous transfection or genomic knock-in)	No (labels endogenous actin directly)
**Isoform Specificity**	Yes (differentiates β-, γ-, or other specific isoforms) [47]	No (pan-actin label)
**Linkage Error**	Low (~4–5 nm; compact fluorophore-conjugated nanobody)	Higher (~15–20 nm; requires primary and secondary antibodies)
**Signal Amplification**	Direct (1:1 or 1:2 nanobody to fluorophore ratio, though highly specific)	High (amplification via multiple secondary antibodies, may give non-specific binding)
**Mammalian Cell Compatibility**	Excellent (validated in U2OS, HT1080, N2a)	Excellent (validated in U2OS, HT1080)
**Primary Neuron Compatibility**	Excellent (superior detection of the ~190 nm periodic MPS rings)	Very good (poor detection of the ~190 nm periodic MPS rings in our study)
**Fungal Model Compatibility**	Excellent (robust post-expansion staining in *S. cerevisiae* and *S. pombe*)	Fails (does not stain yeast actin networks efficiently post-expansion)
**Pre- vs. Post- expansion labeling**	High (post-expansion labeling of denatured epitopes with small nanobody)	Potentially limited (due to pre-expansion HAK-actin labeling)
**Workflow Compatibility**	Compatible with U-ExM	Compatible with U-ExM (may require pre-expansion or specific labeling workflows)

The main advantage of IntAct-U-ExM is demonstrated by the versatile nature of the internal ALFA tag, combined with the compact size of the ALFA nanobody (NbALFA) [48]. These physical properties make the system ideally suited for super-resolution microscopy, as it minimizes the linkage error as compared to large antibodies. Besides its application for IntAct, our study establishes the ALFA tag-NbALFA pair as a highly effective epitope-probe system for the detection of other targets in U-ExM, highlighting its broader utility for diverse biological applications. Beyond actin, this internal tagging strategy holds significant promise for adaptation in divergent, non-model species where conventional antibodies show poor cross-reactivity. It also provides a vital blueprint for labeling other essential, structurally sensitive proteins where traditional N- or C-terminal tagging disrupts endogenous function or fails to endure the stringent denaturation steps of the ExM workflow.

Experiments from our previous [47] and current study strongly suggest that IntAct incorporates very well into native actin filament networks and preserves normal actin network dynamics and function when expressed at native levels. However, it cannot be completely ruled out that IntAct expression may cause abnormal effects in cells, as increased actin expression can perturb normal actin homeostasis [74] and functional checks must be done on a case-by-case basis to avoid any artifacts. This raises another important question: Do IntAct-containing actin filaments behave similarly to native actin filaments under various perturbations? We have tested IntAct-U-ExM with several routinely-used drugs/chemical inhibitors (such as CK666, Latrunculin B, Blebbistatin, Jasplakinolide) which exert different effects on the actin cytoskeleton. IntAct-U-ExM showed compatibility with all tested inhibitors and recapitulated their known phenotypic effects on the actin cytoskeleton across species. This compatibility with diverse experimental regimes establishes IntAct-U-ExM as a functional method for high-resolution analysis of cytoskeletal regulation across diverse model systems.

Going forward, IntAct-U-ExM has tremendous application potential due to the rapid, ongoing technological advancements in ExM, such as the development of novel hydrogel chemistries that allow for increased expansion factors [81] (e.g., iterative expansion [23]) and improved preservation by cryo-fixation [45]. Additionally, workflows combining ExM with other super-resolution modalities, like SIM [28], STED [27], and SMLM [26,82] provide an ideal next step to move toward molecular resolution [29]. Ultimately, IntAct-U-ExM could reveal many new fundamental insights into the nanoscale architecture and functional divergence of actin isoforms and networks across the tree of life.

## Materials and methods

### Plasmids and yeast strains used in the study

All plasmids and yeast strains used in this study are described in S1 and S2 Tables, respectively. The plasmids are available through Addgene (details in S1 Table).

### Yeast growth

*S. cerevisiae* and *S. pombe* strains were grown overnight in Synthetic Complete media lacking uracil (SC-ura) at 25 °C with shaking at 250 rpm. The overnight culture was diluted to a O.D_600_ = 0.2, grown till mid-log phase and harvested for immunofluorescence or ExM.

### Cell culture and transfection

#### U2OS cells.

U2OS (ATCC HTB-96) cells were cultured under standard conditions at 37 °C in a humidified 5% CO_2_ incubator in Dulbecco’s Modified Eagle’s Medium (DMEM, Gibco 11965118) containing 10% FBS (Gibco A5256701), 100 U/mL Penicillin, 0.1 mg/mL Streptomycin (Gibco 15140-122), and 2 mM L-glutamine (HiMedia, TCL012) in a humidified incubator. For transfections, 4 × 10^4^ cells/500 µl were plated on 12-mm glass coverslips in a 24-well plate, cultured overnight and then, transfected with 1 µg of pcDNA3.1-β-IntAct or pcDNA3.1-γ-IntAct with jetPEI (PolyPlus) transfection medium following the manufacturer’s instructions. Twenty-four hours post-transfection, the culture media was aspirated, and the cells were fixed with 4% paraformaldehyde in phosphate-buffered saline (PBS), pH 7.2 for 15 min at room temperature (RT) followed by washing with PBS. The fixed samples were stored at 4 °C till further use.

#### N2a cells.

Neuro-2a cell line, a mouse neuroblastoma cell line (ATCC CCL-131TM, RRID: CVCL_0470) was cultured at 37 °C and 5% CO_2_ in Dulbecco modified Eagle medium (Gibco 12430-054) with 100 U Penicillin/mL and 0.1 mg Streptomycin/mL (Pen Strep Gibco 15140-122), 10% Fetal Bovine Serum (FBS; HIMEDIA RM10434), and 1% Glutamax (Gibco 35050/061). For transfection, 6 × 10^4^ cells/well were seeded on 18-mm coverslips in a 12-well plate and grown overnight in complete DMEM after which, the media was changed to pre-filtered DMEM without antibiotics. The cells were transfected with 20 ng of either β-IntAct or γ-IntAct using Lipofectamine 3000 Transfection Reagent (#L3000075, Thermo Scientific) where the plasmid DNA was diluted in 50 µL of Opti MEM Reduced Serum Medium (#31985070, Thermo Scientific) along with 1 µL of P3000 reagent. Separately, 2 µL of Lipofectamine 3000 reagent was diluted in 50 µL of Opti MEM. The two mixtures were then combined, gently mixed, and incubated at RT for 20 min followed by addition to the cells after which the plate was gently rocked to ensure even distribution. Twenty-four to forty-eight hours post-transfection, the cells were fixed with 4% PFA in PBS for 15 min at RT, washed with PBS, and stored at 4 °C till required for expansion.

#### HT1080.

Human fibrosarcoma HT1080 cells were cultured in 1× DMEM + 4.5 g/L D-Glucose, NEAA (Gibco, Lot#2246377), and supplemented with 10% (vol/vol) fetal bovine serum (FBS), 1× Glutamax (Gibco, 2063631), 1 mM Sodium Pyruvate (Gibco, 2010382), and 0.5× Antibiotic-Antimycotic (Gibco, 15240–062). Cells were incubated at 37 °C and 5% CO_2_.

#### Primary mouse hippocampal neurons.

Primary neurons were cultured from the hippocampi of post-natal Sprague Dawley (SD) rat pups of either sex aged between post-natal day 0 or 1 (P0-P1). Briefly, both hippocampi were dissected from each pup in the Hibernate (Hibernate, Thermofisher) media under a dissection microscope. Cells were dissociated with 0.25% trypsin solution at 37 °C for 10 min followed by washing and mechanical trituration in Hibernate medium supplemented with 1%–2% B-27 (Gibco) and 0.25%–1% Glutamax (Gibco). The single cell suspension was then centrifuged and resuspended on Neurobasal Medium supplemented with 1%–2% B-27, 0.25%–1% GlutaMAX, and 100–200 µg/ml Normocin, and was plated on flatness corrected 18 mm round glass coverslips (VWR) coated with poly-L-lysine (Sigma) (0.1 mg/ml in water). Neurons were maintained for 14–21 days.

#### Nucleofection of primary hippocampal neurons.

Solution I (200 mg ATP disodium salt, 120 mg MgCl_2_·6H_2_O in 1 mL H_2_O) and Solution II (600 mg KH_2_PO_4_, 60 mg NaHCO_3_, 200 mg glucose in 50 mL H_2_O, pH 7.4) were prepared, filter-sterilized (0.22 μm), aliquoted (8 and 400 μL, respectively), and stored at −20 °C. Aliquots were thawed and brought to room temperature before use. Following trituration, dissociated rat hippocampal neurons were resuspended in Hibernate medium, and 4–5 × 10^6^ cells were centrifuged at 80*g* for 5 min at room temperature. The pellet was gently resuspended in 102 μL nucleofector solution (2 μL Solution I + 100 μL Solution II), and 1–3 μg plasmid DNA was added. The suspension was transferred to a nucleofection cuvette (without air bubbles) and electroporated using program G-013 (Lonza). Immediately after nucleofection, 500 μL Neurobasal medium was added, and cells were seeded onto PLL-coated coverslips (12-well plates) at a density of 3 × 10^5^ cells per coverslip. Medium was replaced after 30 min and again at 24 h.

### Ultrastructure expansion microscopy (U-ExM) workflow for yeast

#### Reagents required.

- Acrylamide (AA).

Stock: 40% (Sigma-Aldrich A4058). Stored at 4 °C.

- Formaldehyde (FA).

Stock: 36.5%–38% (Sigma-Aldrich F8775). Stored in the fume hood.

- N, N′-methylenebisacrylamide (BIS).

Stock: 2% (Sigma-Aldrich M1533). Stored at 4 °C.

- Poly-L-lysine.

(Sigma-Aldrich A-003-E). Stored at 4 °C.

- Nuclease-free water.
- Sodium acrylate (SA).

Stock: 97%–99% (Sigma-Aldrich 408220)

- Ammonium persulfate (APS)
- Tetramethylethylenediamine (TEMED)

#### Day 1: Sample preparation and anchoring.

A modified version of a previously described U-ExM protocol for yeast was followed [56]. Yeast cells grown till mid-log phase were fixed with either (i) 4% FA or (ii) 4% FA + 0.1% GA for 15 min in PEM buffer (100 mM PIPES, 1 mM EGTA, 1 mM MgSO_4_, pH adjusted to 6.9). Cells were then washed with 1× PBS three times and then incubated with 100mL of PEM-S (1.2 M Sorbitol in PEM) buffer containing 15 mL of Long-Life Zymolase (#786–036, GBioSciences) at 37 °C for 30–45 min. The cells were then washed twice with PEM-S buffer, resuspended in freshly made 100 mL AA–FA mixture (1%AA + 0.7%FA) made in 1× PBS, and incubated overnight at 37 °C, 350 rpm.

#### Day 2: Seeding, cross-linking to gel, denaturation, and staining.

The overnight incubated mixture of cells was seeded on a 12 mm round glass coverslip coated with poly-L-lysine and allowed to sit for 20 min at room temperature. The excess cell mixture was removed and stored at 4 °C. Simultaneously, 36 mL of monomer solution (MS; 19% (wt/wt) SA, 10% (wt/wt) AA, 0.1% (wt/wt) BIS in PBS) was mixed with 2 mL of 10% TEMED and 2 mL of 10% APS. The mixture was immediately poured as a droplet of 36 mL on a parafilm attached to a flat metal block kept on ice. The coverslip with seeded cells was gently placed on top of the droplet such as to cover the whole droplet and allowed to sit on the cold metal block for 5 min. The metal block was then transferred to a sealed humid chamber and kept at 37 °C for 45 min to allow the polymerization of the gel. The gels were then separated from the coverslips and incubated in denaturation buffer (50 mM Tris pH 9.0, 200 mM NaCl, 200 mM SDS, adjust pH to 9.0 with HCl) for 90 min at 95 °C. This was followed by washing with PBS thrice for 5 min at room temperature. Furthermore, the gels were then stained with FluoTag-X2 anti-ALFA-Alexa647 (#N1502, Nanotag Biotechnologies, 1:100 dilution) overnight in 3% bovine serum albumin (BSA) in 1× PBS-T at 4 °C.

#### Day 3: Imaging.

The gels were stained with DAPI (1:200) for 30 min at room temperature, followed by three washes with 1× PBS-T. Subsequently, the gels were expanded in ddH_2_O thrice for 30 min each and then mounted on a 35 mm glass-bottom dish (Ibidi, Cat.No: 81151) coated with poly-L-lysine. Imaging was done with an Olympus SpinSR spinning disk confocal microscope using a 60× oil-immersion objective (N.A. = 1.42). The samples were excited with a solid-state laser of wavelength 640 nm and a LED light source of wavelength 405nm. The images were acquired with a Prime BSI scMOS camera and deconvolved using Olympus CellSens Dimension software.

### Immunofluorescence imaging of non-expanded yeast cells

Immunofluorescence was performed as described previously [83]. Briefly, yeast strains were grown overnight at 25 °C in YPD broth/EMM-Ura. The overnight culture was diluted and allowed to grow until mid-log phase. Cells were fixed with 4% FA for 60 min at 25 °C, washed twice with 1× PBS, and finally resuspended in 200 μl of 1.2M Sorbitol Phosphate-Citrate (SPC) buffer (1.2 M Sorbitol, 1 M K2HPO4, 1 M Citric acid); 25 μl of Long-Life Zymolase (#786–036, GBiosciences) was added to digest the yeast cell wall and the suspension was incubated with mild shaking at 37 °C for 60 min. The cells were then washed twice with ice-cold SPC buffer and incubated with 500 μl of blocking buffer 2% BSA + 0.1% Triton X-100 in PBS at room temperature for 15 min with shaking. The cells were pelleted and resuspended in 500 μl of Antibody Dilution Buffer (1% BSA + 0.05% Triton X-100 in PBS) containing FluoTag-X2 anti-ALFA-Alexa647 (#N1502, Nanotag Biotechnologies) at a final dilution of 1:500. The cell suspension was then incubated overnight with rotation at 4 °C. Next day, cells were washed twice with 1× PBS and mounted on a glass-bottom dish coated by poly-L-lysine (#P4707, Sigma Aldrich). Phalloidin staining of yeast actin structures was done as per previously described protocols [84]. Briefly, cells were grown at 25 °C till mid-log phase and fixed with 4% paraformaldehyde. The cells were washed thrice with 1× PBS, and labeled phalloidin was added to a final concentration of 0.4 μM (in 50 μl of 1× PBS), and the tubes were kept in a rotating shaker overnight at 4 °C. The cells were washed twice with 1× PBS on the next day and seeded on a concanavalin A-coated glass-bottom dish. Imaging was done with an Olympus SpinSR spinning disk confocal microscope using a 100× oil-immersion objective (N.A. = 1.45), equipped with solid-state lasers of wavelength 640 and 488 nm, and a LED light source of wavelength 405 nm. The images were acquired with a Prime BSI scMOS camera and deconvolved using Olympus CellSens Dimension software.

### Ultrastructure expansion microscopy (U-ExM) workflow for U2OS and HT1080 cells

#### Materials required.

- Nuclease-free water (NFW, #AM9937, Ambion-ThermoFisher)
- Poly-D-Lysine (#A3890401, Gibco)
- Ammonium persulfate (APS, #17874, ThermoFisher)
- Formaldehyde (FA, #F8775, SIGMA)
- Tetramethylethylenediamine (TEMED, #17919, ThermoFisher)
- Acrylamide (AA, 40%, #A4058, SIGMA)—Ready to use—Keep at 4 °C
- N, N’-methylenebisacrylamide (BIS, 2%, #M1533, SIGMA)
- Sodium Acrylate (SA, #408220, SIGMA)
- Glass-bottom Confocal dish (#BDD-002-035, BioFil)

#### Day 1: Fixing and first round of expansion.

The β-IntAct or γ-IntAct transfected U2OS cells, fixed with 4% PFA were first incubated with 2.8% FA / 5% acrylamide solution for 5 h at 37 °C. Then, the solution was removed, and the cells were incubated with 35 µL of monomer solution (composed of 25% acrylamide, 5% bis-acrylamide, 19% sodium acrylate, 0.5% TEMED, and 0.5% ammonium persulfate) for 5 min on ice, followed by 1 h at 37 °C to allow gelation. Post gelation, the coverslips with the gels were transferred to a 6-well plate and incubated with 1 mL of denaturation buffer (50 mM Tris/HCl, pH 9.0 containing 200 mM SDS and 200 mM NaCl) for 15 min at RT with shaking. Once the gel detached from the coverslip, it was tranferred carefully with a spatula into a fresh 1.5mL vial filled with 1–2 mL of fresh denaturation buffer and incubated for 90 min at 95 °C. Post-denaturation, the gel was carefully transferred to a glass petri-dish with distilled water for 30 min followed by changing the water and incubating the gels overnight for complete expansion.

#### Day 2: Staining, expansion, and imaging.

The expanded gels were exchanged from water to 1xPBS twice for 15 min each to shrink the gel for immunostaining. Then, a small piece of the gel was cut using a scalpel and carefully transferred to a 24-well plate and incubated with Phalloidin-Alexa488 (#49409, Sigma Aldrich) at a final dilution of 1:50 and FluoTag-X2 anti-ALFA-Alexa647/Atto488 (#N1502, Nanotag Biotechnologies) at a final dilution of 1:500, both diluted in PBS containing 2% BSA for 2 h 30 min at 37 °C. The gels were subsequently washed 3 times with PBS containing 0.1% Tween 20 (PBST) for 10 min at RT with agitation. Then, the gel was incubated with DAPI (#D1306, Thermo Scientific) at a final dilution of 1:200 in PBS for 20 min RT with agitation following which, the gel piece was transferred to a glass petri-dish containing distilled water for 30 min for the final round of expansion before proceeding to image the gels. Once expanded, a small piece of the expanded gel was decanted of any excess water by gently tapping it with tissue paper. and transferred to a poly-D-lysine-coated 35 mm glass-bottom confocal dish and gently pressed it to ensure it is adhered to the dish surface. The piece was then imaged using a 63× oil immersion objective (N.A. = 1.42) on the Zeiss Axio Observer 7 epifluorescence microscope equipped with the Orca-Flash4.0 V3 sCMOS camera (Hamamatsu) using the ZEN 3.8 (Carl Zeiss) software or Olympus SpinSR spinning disk confocal microscope using a 60× oil-immersion objective (N.A. = 1.42) using the Prime BSI sCMOS camera and CellSens (Olympus) software. The images were deconvolved using the deconvolution modules within the respective software when required. HT1080 cells were imaged using Zeiss LSM880 with Airyscan, and data was acquired using a 63× 1.4 NA oil objective.

### Immunofluorescence imaging of non-expanded U2OS and N2a cells

The β-IntAct or γ-IntAct transfected U2OS cells, fixed with 4% PFA were incubated with Phalloidin-Alexa488 (1:200 dilution), FluoTag-X2 anti-ALFA-Alexa647 (1:500 dilution), and DAPI (1:1,000 dilution) all diluted in PBS containing 2% BSA for 2 h at RT. The coverslips were washed 3 times with PBST for 3 min at RT and mounted onto a glass slide using ProLong Gold (#P36934, Thermo Scientific) and allowed to polymerize overnight at RT. The cells were imaged using a 63× oil-immersion objective (N.A. = 1.42) on the Zeiss Axio Observer 7 epifluorescence microscope equipped with the Orca‑Flash4.0 V3 sCMOS camera (Hamamatsu) using the ZEN 3.8 (Carl Zeiss) software

### Ultrastructure expansion microscopy (U-ExM) workflow for N2a cells and primary mouse hippocampal neurons

Neuro-2a cells and primary mouse neurons were processed for U-ExM using the same protocol as mentioned above for yeast cells.

### HAK-actin treatment and staining

For HAK-actin staining, cells (*S. cerevisiae*, *S. pombe*, U2OS, HT1080, primary mouse hippocampal neurons) were fixed with 4% FA + 0.1% GA for 15 min at room temperature. Fixed cells were then incubated with HAK-actin staining solution (3% BSA in 1× PBS-T) at 1:500 dilution for 2 h at room temperature, followed by two mild washes in 1× PBS-T. Then, the cells were again post-fixed with 4% FA + 0.0125% GA at room temperature for 10 min. The cells were then washed three times with 1× PBS, followed by anchoring in a solution of acrylamide and formaldehyde (AA + FA), based on the U-ExM protocols mentioned above. All the subsequent steps were followed as listed above in the U-ExM protocols for the different species. For staining, anti-HA antibody (Santa Cruz/#sc-7392/Lot.No. L1820) was used at a concentration of 1:500.

### Chemical inhibitor treatments

The following chemical inhibitors (Table 2) were used in Fig 4:

### Image analysis

All images were analyzed in Fiji (ImageJ). The nuclei were segmented using automatic segmentation using the Labkit plug-in. The cell dimensions were measured manually in ImageJ. Expansion factors were calculated by comparing measured parameters (nucleus area, cell area, cell length, and width) between non-expanded and expanded cells. For FWHM analysis, line plots perpendicular to the yeast actin cables were generated with ImageJ and non-linear gaussian curve fitting was done in GraphPad Prism (v10.2) using the equation:


$$\begin{array}{c} Y\;=\;a\;+\;(b\;-\;a)\;*\text{ exp}(-\;({\left(x\;-\;c\right)}^{\text{2}})/(\text{2}\;*\;d^{\text{2}})) \end{array}$$

where amplitude = (*b* − a); mean = *c*; Standard deviation (SD) = *d*; Baseline = *a* (*Y* at *x* = 0). The FWHM was calculated as (2.355 * SD), and the values were plotted using GraphPad Prism (v10.2). FWHM values were scaled with the average expansion factor for budding yeast and fission yeast.

### Measurement of distance between neuronal actin rings

Actin line scan intensity profiles were analyzed using a custom Python-based pipeline to quantify spatial periodicity. Line scan data containing intensity values and corresponding spatial positions (in microns) were processed in batch mode. Peaks were detected using the scipy.signal.find_peaks function with constraints on minimum peak separation and prominence to minimize noise-driven detections. Peak-to-peak distances were calculated from the spatial positions of consecutive peaks to estimate periodic spacing. For each line scan, distributions of peak spacing were generated, and summary statistics were computed. Spacing values from all samples were further pooled to obtain a global distribution. All analyses were performed using Python (NumPy, SciPy, Pandas, and Matplotlib), and the complete analysis script is provided as a supplementary file S1 Script.

## Supporting information

S1 Fig **(A)** Representative maximum intensity projected images showing comparison of expanded *Saccharomyces cerevisiae* cells fixed with either 4% FA (top row) or 4% FA + 0.1% GA (bottom row), stained with NbALFA-Alexa647. **(B)** Representative maximum intensity projected images showing comparison of expanded *Schizosaccharomyces pombe* cells fixed with either 4% FA (top row) or 4% FA + 0.1% GA (bottom row), stained with NbALFA-Alexa647. **(C, D)** Plots depicting measurements of Full Width at Half Maxima (FWHM) for actin cables in non-expanded (phalloidin-stained) and expanded (NbALFA-Alexa647-stained) *S. cerevisiae* (C) (*n* ≥ 32 measurements for all categories) and *S. pombe* (D) (*n* ≥ 41 measurements for all categories); values from expanded samples were scaled down with the average expansion factor of 4.92 for *S.c.* and 4.58 for *S.p.* (E) Plot depicting measured actomyosin ring diameter in expanded *S. cerevisiae* (*n* ≥ 13 rings) and *S. pombe* cells (*n* ≥ 11 rings). The numerical data underlying this figure can be found in S1 Data.
(TIFF)

S2 Fig **(A)** Plots representing measurements of nucleus area in non-expanded and expanded U2OS cells expressing β-IntAct or γ-IntAct along with calculated expansion factors (*n* ≥ 32 cells for β-IntAct, *n* ≥ 16 for γ-IntAct). **(B)** Representative maximum intensity projected images qualitatively showing difference in resolution across non-expanded and expanded human U2OS cells expressing IntAct and stained as indicated. **(C)** Representative single plane or maximum intensity projected images of expanded human U2OS cells expressing either β- or γ-IntAct stained with NbALFA-Alexa647 showing presence of actin filaments inside the nuclear volume. **(D)** Plots representing measurements of nucleus area in non-expanded and expanded HT1080 cells expressing β-IntAct or γ-IntAct along with calculated expansion factors (*n* ≥ 33 cells for β-IntAct, *n* ≥ 18 for γ-IntAct). The numerical data underlying this figure can be found in S1 Data.
(TIFF)

S3 Fig **(A)** Representative maximum intensity projected images of non-expanded and expanded mouse Neuro-2a (N2a) cells expressing β-IntAct stained as indicated. **(B)** Representative maximum intensity projected images of non-expanded and expanded mouse Neuro-2a (N2a) cells expressing γ-IntAct stained as indicated. **(C)** Plots representing measurements of nucleus area in non-expanded and expanded mouse N2a cells expressing β-IntAct or γ-IntAct along with calculated expansion factors (*n* ≥ 23 cells for β-IntAct, *n* ≥ 26 for γ-IntAct). **(D)** Representative maximum intensity projected images of expanded human mouse N2a cells expressing either β- or γ-IntAct stained with NbALFA-Alexa647 showing various actin structures. **(E)** Representative images of non-expanded primary mouse hippocampal neurons (div14) expressing β-IntAct or γ-IntAct stained for F-actin (phalloidin), IntAct (NbALFA-Alexa647) and DNA (DAPI). The numerical data underlying this figure can be found in S1 Data.
(TIFF)

S4 Fig **(A)** Representative maximum intensity projected images of expanded HT1080 cells natively expressing either β- or γ-IntAct stained with NbALFA-Ab635p and HAK-actin. **(B)** Representative maximum intensity projected images of expanded U2OS cells exogenously expressing either β- or γ-IntAct stained with NbALFA-Ab635p and HAK-actin. **(C)** Representative maximum intensity projected images of expanded primary mouse hippocampal neurons (div14) expressing either β- or γ-IntAct stained with NbALFA-Ab635p and HAK-actin. **(D)** Representative maximum intensity projected images of expanded *Saccharomyces cerevisiae* and *Schizosaccharomyces pombe* cells expressing IntAct stained with NbALFA-Ab635p and HAK-actin.
(TIFF)

S5 Fig Schematic representation of the IntAct-U-ExM workflow. The IntAct probe, containing an ALFA tag, is expressed across different model systems where it successfully incorporates into native actin filaments alongside endogenous actin monomers. The cells are then subjected to the Ultrastructural Expansion Microscopy (U-ExM) protocol, which involves fixation, gel embedding, denaturation, and physical expansion. Finally, the expanded actin networks are stained for the ALFA tag and imaged, enabling super-resolution visualization of the actin cytoskeleton. This figure illustration was created using BioRender. Palani, S. (2025) https://BioRender.com/vgn12tr.
(PNG)

S1 Movie Movie representing 3D volume (z-stacks) of expanded *Saccharomyces cerevisiae* and *Schizosaccharomyces pombe* cells stained for IntAct with NbALFA-Alexa647 (blue). (AVI)

S2 Movie Movie representing 3D volume (z-stacks) of expanded *Schizosaccharomyces pombe* cells treated with Arp2/3 inhibitor CK666 stained for IntAct with NbALFA-Alexa647 (orange). Different colors represent different z-depth.
(AVI)

S3 Movie Movie representing 3D volume (z-stacks) of expanded U2OS cells stained for β- or γ-IntAct with NbALFA-Alexa647 (orange). (AVI)

S1 Table List of plasmids used in this study. (DOCX)

S2 Table List of yeast strains used in this study. (DOCX)

S1 Script Pipeline for measurement of Actin Ring Spacing in neurons. (DOCX)

S1 Data Excel spreadsheet with individual numerical data underlying plots organized into separate sheets corresponding to the following figure panels: 1C, 1D, 3C, 3F, S1C, S1D, S1E, S2A, S2D, and S3C. (XLSX)

## Abbreviations

AA: Acrylamide
BSA: bovine serum albumin
ExM: expansion microscopy
FA: formaldehyde
GA: glutaraldehyde
LatB: Latrunculin B
MPS: Membrane-associated Periodic Cytoskeleton
PBS: phosphate-buffered saline
PFA: paraformaldehyde
RT: room temperature
SA: Sodium acrylate
S.c.: Saccharomyces cerevisiae
S.p.: Schizosaccharomyces pombe
SD: Sprague Dawley
U-ExM: ultrastructure expansion microscopy
