# Supplementary figures and images for: IntAct-U-ExM enables super-resolution imaging of isoform-specific actin networks across species
Source: PLoS Biol. 2026 Jun 12;24(6):e3003832. doi: 10.1371/journal.pbio.3003832 (PMC13262867; doi:10.1371/journal.pbio.3003832)

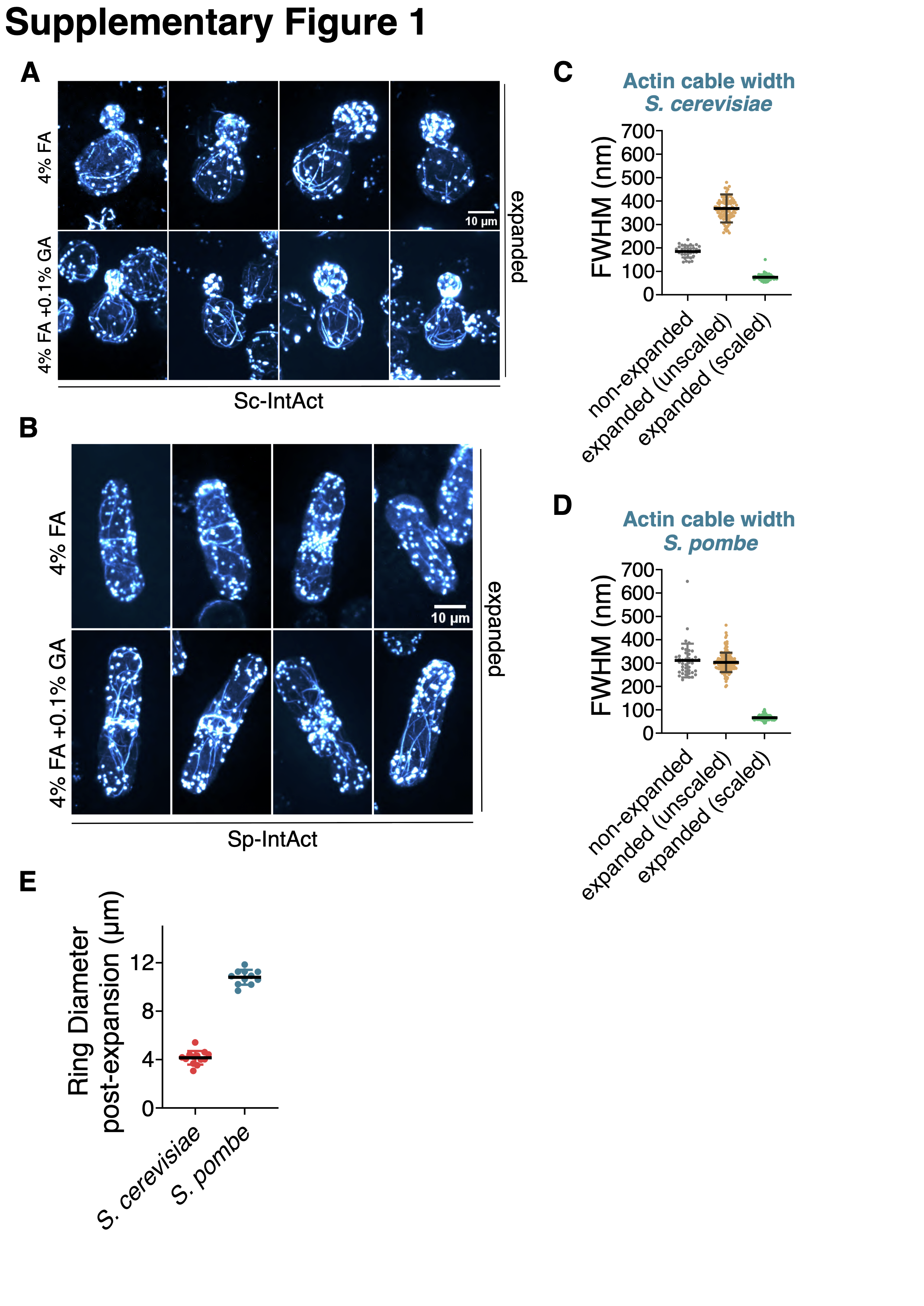

Supplement: S1 Fig — (A) Representative maximum intensity projected images showing comparison of expanded Saccharomyces cerevisiae cells fixed with either 4% FA (top row) or 4% FA + 0.1% GA (bottom row), stained with NbALFA-Alexa647. (B) Representative maximum intensity projected images showing comparison of expanded Schizosaccharomyces pombe cells fixed with either 4% FA (top row) or 4% FA + 0.1% GA (bottom row), stained with NbALFA-Alexa647. (C, D) Plots depicting measurements of Full Width at Half Maxima (FWHM) for actin cables in non-expanded (phalloidin-stained) and expanded (NbALFA-Alexa647-stained) S. cerevisiae (C) (n ≥ 32 measurements for all categories) and S. pombe (D) (n ≥ 41 measurements for all categories); values from expanded samples were scaled down with the average expansion factor of 4.92 for S.c. and 4.58 for S.p. (E) Plot depicting measured actomyosin ring diameter in expanded S. cerevisiae (n ≥ 13 rings) and S. pombe cells (n ≥ 11 rings). The numerical data underlying this figure can be found in S1 Data. (TIFF) [file pbio.3003832.s001.tiff]

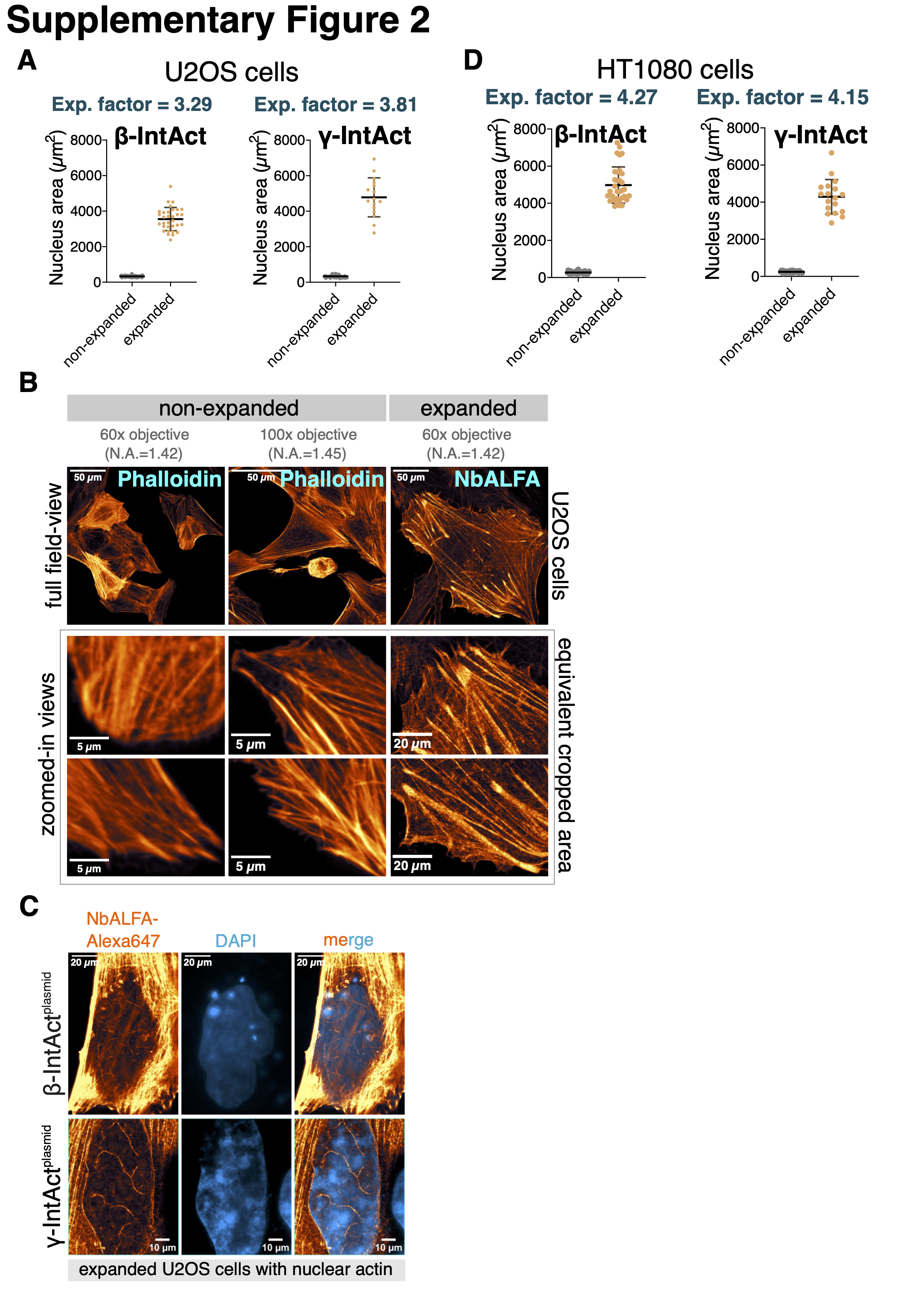

Supplement: S2 Fig — (A) Plots representing measurements of nucleus area in non-expanded and expanded U2OS cells expressing β-IntAct or γ-IntAct along with calculated expansion factors (n ≥ 32 cells for β-IntAct, n ≥ 16 for γ-IntAct). (B) Representative maximum intensity projected images qualitatively showing difference in resolution across non-expanded and expanded human U2OS cells expressing IntAct and stained as indicated. (C) Representative single plane or maximum intensity projected images of expanded human U2OS cells expressing either β- or γ-IntAct stained with NbALFA-Alexa647 showing presence of actin filaments inside the nuclear volume. (D) Plots representing measurements of nucleus area in non-expanded and expanded HT1080 cells expressing β-IntAct or γ-IntAct along with calculated expansion factors (n ≥ 33 cells for β-IntAct, n ≥ 18 for γ-IntAct). The numerical data underlying this figure can be found in S1 Data. (TIFF) [file pbio.3003832.s002.tiff]

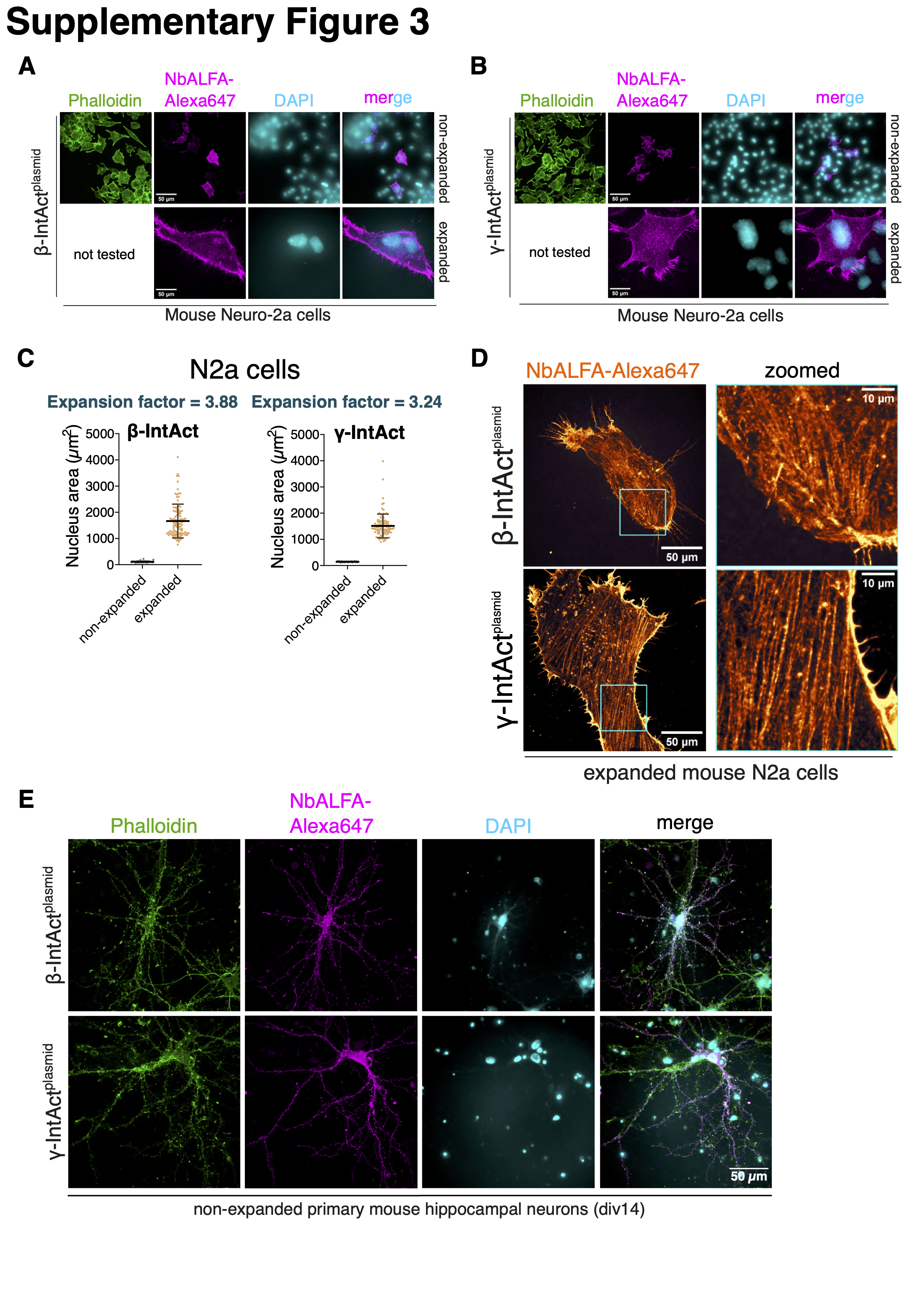

Supplement: S3 Fig — (A) Representative maximum intensity projected images of non-expanded and expanded mouse Neuro-2a (N2a) cells expressing β-IntAct stained as indicated. (B) Representative maximum intensity projected images of non-expanded and expanded mouse Neuro-2a (N2a) cells expressing γ-IntAct stained as indicated. (C) Plots representing measurements of nucleus area in non-expanded and expanded mouse N2a cells expressing β-IntAct or γ-IntAct along with calculated expansion factors (n ≥ 23 cells for β-IntAct, n ≥ 26 for γ-IntAct). (D) Representative maximum intensity projected images of expanded human mouse N2a cells expressing either β- or γ-IntAct stained with NbALFA-Alexa647 showing various actin structures. (E) Representative images of non-expanded primary mouse hippocampal neurons (div14) expressing β-IntAct or γ-IntAct stained for F-actin (phalloidin), IntAct (NbALFA-Alexa647) and DNA (DAPI). The numerical data underlying this figure can be found in S1 Data. (TIFF) [file pbio.3003832.s003.tiff]

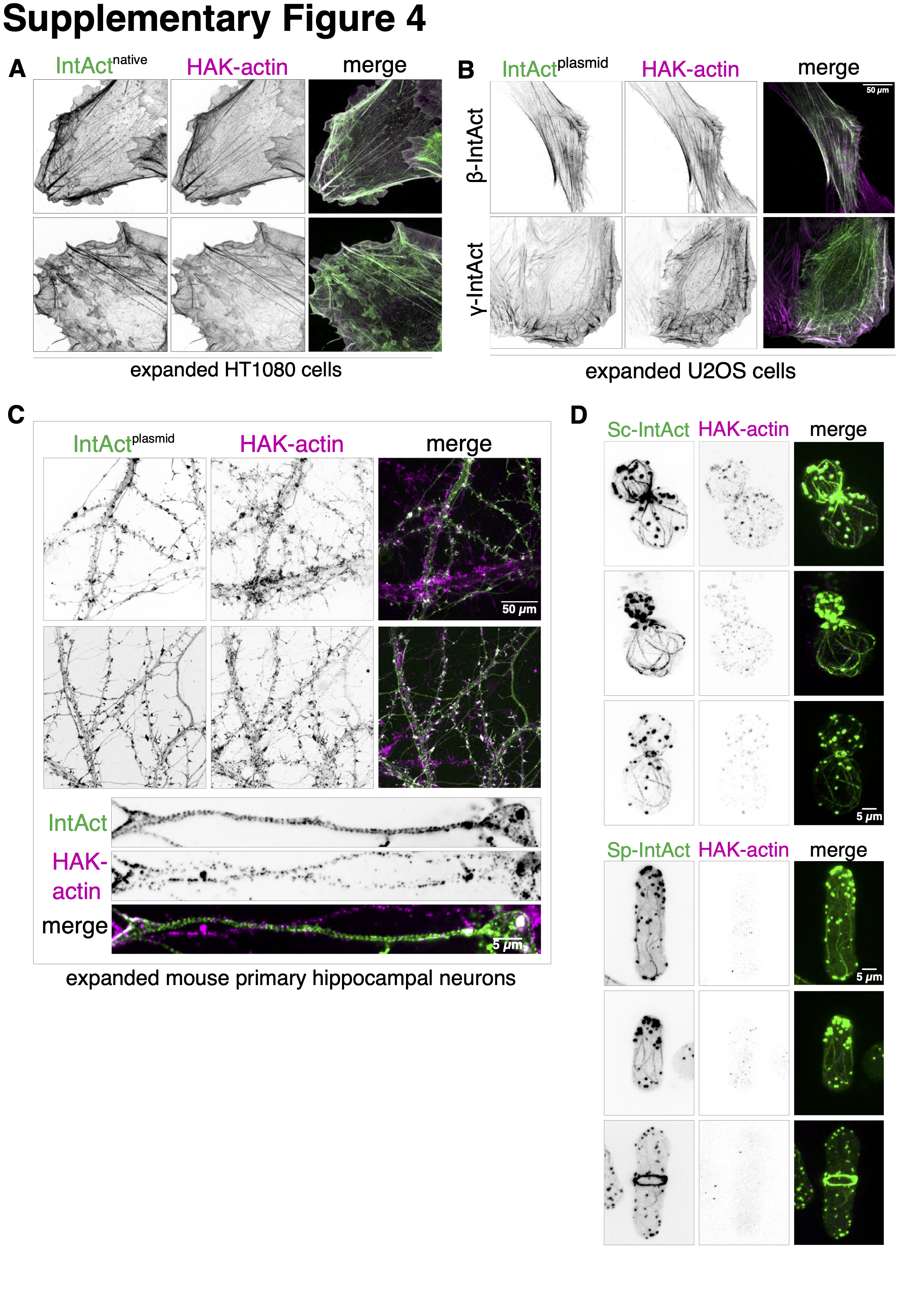

Supplement: S4 Fig — (A) Representative maximum intensity projected images of expanded HT1080 cells natively expressing either β- or γ-IntAct stained with NbALFA-Ab635p and HAK-actin. (B) Representative maximum intensity projected images of expanded U2OS cells exogenously expressing either β- or γ-IntAct stained with NbALFA-Ab635p and HAK-actin. (C) Representative maximum intensity projected images of expanded primary mouse hippocampal neurons (div14) expressing either β- or γ-IntAct stained with NbALFA-Ab635p and HAK-actin. (D) Representative maximum intensity projected images of expanded Saccharomyces cerevisiae and Schizosaccharomyces pombe cells expressing IntAct stained with NbALFA-Ab635p and HAK-actin. (TIFF) [file pbio.3003832.s004.tiff]

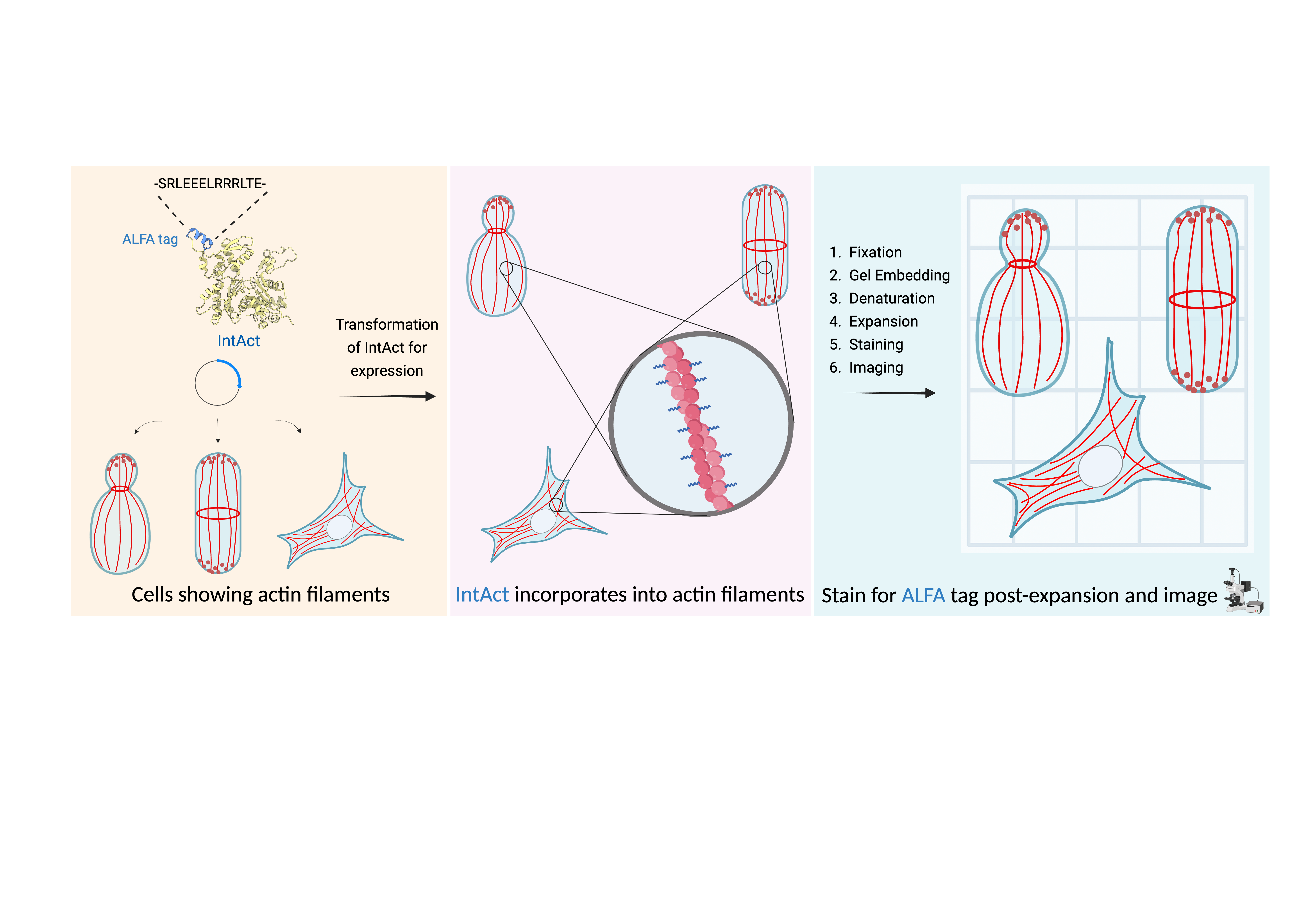

Supplement: S5 Fig — The IntAct probe, containing an ALFA tag, is expressed across different model systems where it successfully incorporates into native actin filaments alongside endogenous actin monomers. The cells are then subjected to the Ultrastructural Expansion Microscopy (U-ExM) protocol, which involves fixation, gel embedding, denaturation, and physical expansion. Finally, the expanded actin networks are stained for the ALFA tag and imaged, enabling super-resolution visualization of the actin cytoskeleton. This figure illustration was created using BioRender. Palani, S. (2025) https://BioRender.com/vgn12tr. (PNG) [file pbio.3003832.s005.png]
