# Supplementary material for: IntAct-U-ExM enables super-resolution imaging of isoform-specific actin networks across species
Source: PLoS Biol. 2026 Jun 12;24(6):e3003832. doi: 10.1371/journal.pbio.3003832 (PMC13262867; doi:10.1371/journal.pbio.3003832)
Supplement: S1 Table — (DOCX) [file pbio.3003832.s009.docx]

**S1 Table. List of plasmids used in this study**

| **Plasmid Number (Addgene ID)** | **Description** | **Source** |
| --- | --- | --- |
| piSP1465 (248980) | pRS316-pTEF-ScIntAct-tCYC | *van Zwam et al. 2024*^47^ |
| piSP1486 (250862) | pDUAL-pAct-SpIntAct-tCYC | *van Zwam et al. 2024*^47^ |
| piSP1596 (250439) | pcDNA3.1(+)-beta-IntAct | *van Zwam et al. 2024*^47^ |
| piSP1597 (250440) | pcDNA3.1(+)-gamma-IntAct | *van Zwam et al. 2024*^47^ |
