# Supplementary material for: IntAct-U-ExM enables super-resolution imaging of isoform-specific actin networks across species
Source: PLoS Biol. 2026 Jun 12;24(6):e3003832. doi: 10.1371/journal.pbio.3003832 (PMC13262867; doi:10.1371/journal.pbio.3003832)
Supplement: S2 Table — (DOCX) [file pbio.3003832.s010.docx]

**S2 Table. List of yeast strains used in this study.**

| **Strain Number** | **Genotype** | **Source** |
| --- | --- | --- |
| YSP641 | *MATa leu2∆1 trp1∆63 his3∆200 ura3-52 :: pRS316-pTEF-Sc-IntAct-tCYC-ura3+* | *van Zwam et al. 2024*^47^ |
| YSP1049 | *ura4-D18 leu1-32 h- :: pDUAL-pAct-Sp-IntAct-Tadh1-ura4+* | *van Zwam et al. 2024*^47^ |
