# Supplementary material for: IntAct-U-ExM enables super-resolution imaging of isoform-specific actin networks across species
Source: PLoS Biol. 2026 Jun 12;24(6):e3003832. doi: 10.1371/journal.pbio.3003832 (PMC13262867; doi:10.1371/journal.pbio.3003832)
Supplement: S1 Script — (DOCX) [file pbio.3003832.s011.docx]

Peak Spacing Analysis Pipeline for Actin Line Scans

# Overview

This pipeline performs automated analysis of line scan intensity profiles to quantify spatial periodicity.

It detects peaks, computes peak-to-peak spacing, and generates both per-sample and aggregated outputs.

# Input Requirements

CSV files must contain:

- intensity: intensity values along the line scan

- position or distance: spatial position in microns (µm)

# Usage

1. Place all CSV files in a single directory.

2. Activate your Python environment.

3. Run: python batch_peak_analysis.py

# Outputs (Per File)

- *_linescan.png: raw line scan

- *_peaks.png: detected peaks overlay

- *_spacing_hist.png: histogram of spacing

- *_peak_spacings.csv: numerical data

# Aggregated Outputs

- merged_peak_spacings.csv: combined spacing data

- merged_spacing_histogram.png: global distribution

# Methodology

Minimal Gaussian smoothing (σ = 0.1) is applied.

Peaks are detected using scipy.signal.find_peaks with distance and prominence constraints.

Spacing is computed as distance between consecutive peaks.

All spacings are aggregated for global statistical analysis.

# Full Script

import numpy as np

import pandas as pd

import matplotlib.pyplot as plt

from scipy.signal import find_peaks

from scipy.ndimage import gaussian_filter1d

import os

import glob

input_folder = r"E:\AD_directory\line_scan_csv"

expected_spacing = 1.0

csv_files = glob.glob(os.path.join(input_folder, "*.csv"))

all_spacings = []

all_labels = []

for file in csv_files:

base_name = os.path.splitext(os.path.basename(file))[0]

df = pd.read_csv(file)

df.columns = df.columns.str.strip()

if "intensity" not in df.columns:

continue

I = df["intensity"].values

for col in df.columns:

if "pos" in col.lower() or "dist" in col.lower():

x = df[col].values

break

dx = np.mean(np.diff(x))

I_smooth = gaussian_filter1d(I, sigma=0.1)

min_distance = max(1, int(0.5 * expected_spacing / dx))

peaks, _ = find_peaks(I_smooth, distance=min_distance, prominence=10)

peak_positions = x[peaks]

spacings = np.diff(peak_positions)

if len(spacings) > 0:

all_spacings.extend(spacings)

all_labels.extend([base_name] * len(spacings))
