# Supplementary material for: IntAct-U-ExM enables super-resolution imaging of isoform-specific actin networks across species
Source: PLoS Biol. 2026 Jun 12;24(6):e3003832. doi: 10.1371/journal.pbio.3003832 (PMC13262867; doi:10.1371/journal.pbio.3003832)
Supplement: Attachment — Submitted filename: Response_to_Reviewers_PBIOLOGY-D-25-02426R3.pdf [file pbio.3003832.s014.pdf]

Response to Reviewers' Comments

Reviewer remarks:

Reviewer #1: The authors addressed my concerns and made an honest effort to address the other reviewers concerns as well. I agree to proceed with publication.

We thank the reviewer for the positive evaluation and recommendation for publication.

Reviewer #2: The authors have compellingly addressed all the points raised.

We thank the reviewer for the positive evaluation and confirmation that our revisions have satisfactorily addressed the concerns raised.

Reviewer #3: The manuscript is technically strong, clearly written, and presents a useful extension of existing imaging approaches, but in its current form it still does not quite meet the threshold for PLOS Biology. The central limitation is one of scope: rather than delivering a major biological insight, the study primarily represents a well-executed extension of an already published tool. While it convincingly demonstrates that IntAct can be adapted to U-ExM across multiple systems, the overall contribution reads more as method optimization than as a broad conceptual advance. This impression is reinforced by several points. First, the work builds directly on the prior IntAct study, with the main novelty arising from its combination with U-ExM rather than from the introduction of a fundamentally new principle. Second, although the revised manuscript includes useful additions such as comparisons to HAK-actin and application to primary neurons, much of the data still lacks quantitation (including the comparison to HAK-actin, making it completely subjective) and does not present any clearly novel biological findings that are uniquely enabled by this approach. Overall, the manuscript is solid and publishable, and will likely be valuable to the field, but it appears better suited for an imaging- or methods-focused journal.

We thank the reviewer for the careful assessment and for acknowledging that the manuscript is technically strong, clearly written, and will be valuable to the field.

Reviewer #4: The revised manuscript has substantially improved. Residual concerns are minor and can be resolved with text revisions only.

We thank the reviewer for the positive overall assessment and for the constructive suggestions, which we have addressed below.

Minor concern:

The median ring spacing of 975 nm for β-IntAct slightly exceeds the expected 5× expansion upper bound of 950 nm, and differs non-trivially from the 866 nm observed for γ-IntAct. The authors should add 1-2 sentences in the Results (around Lines 227-231) explaining whether this isoform

difference is expected or reflects measurement scatter, and whether the slight exceedance of the 5× bound is within the variability of local expansion factors.

We thank the reviewer for raising this point. The higher median ring spacing observed for β-IntAct is most likely a result of measurement scatter or variation in expansion factors or a combination of both, and is not as such expected since, there is no prior work to suggest any kind of difference between β-actin and γ-actin. Thus, more careful evaluation and corroboration in the future with other techniques such as SMLM or STED would be required to further assess whether any isoform-differences exist. As suggested, we have now clarified this in the revised manuscript in the Results section **(Lines 235-242)**.

Each instance in the manuscript where 'isoform-specific' imaging is claimed should be paired with a clear reference to van Zwam et al. (2024) as the source of isoform distribution validation. This ensures readers understand that the specificity claim rests on documented prior work rather than direct demonstration in the current study.

We thank the reviewer for this helpful suggestion, which strengthens the rigor of our specificity claims. We have systematically reviewed the manuscript and added citations to *van Zwam et al.* (*2024*) at every location where isoform-specific labelling is claimed **(Lines 193, 247, 321, and 343)**, making clear that the specificity claim rests on the documented validation of the IntAct strategy in our previous work.

References

1. Zwam, M.C. van, A. Dhar, W. Bosman, W. van Straaten, S. Weijers, E. Seta, B. Joosten, J. van Haren, S. Palani, and K. van den Dries. 2024. IntAct: A nondisruptive internal tagging strategy to study the organization and function of actin isoforms. *PLOS Biology*. 22:e3002551. doi:10.1371/journal.pbio.3002551.
