# Supplementary material for: IntAct-U-ExM enables super-resolution imaging of isoform-specific actin networks across species
Source: PLoS Biol. 2026 Jun 12;24(6):e3003832. doi: 10.1371/journal.pbio.3003832 (PMC13262867; doi:10.1371/journal.pbio.3003832)
Supplement: Peer review history

# Editor report: Decision Letter 0

**DOI:** 10.1371/journal.pbio.3003832.r001

**Authors:** Richard Hodge

12 Aug 2025

Dear Dr Palani,

Thank you for submitting your manuscript entitled "IntAct-U-ExM: Ultrastructure Expansion microscopy of actin networks via an internally-tagged actin" for consideration as a Update Article by PLOS Biology. Please accept my sincere apologies for the delay in getting back to you as we consulted with an academic editor about your submission.

Your manuscript has now been evaluated by the PLOS Biology editorial staff, as well as by an academic editor with relevant expertise, and I am writing to let you know that we would like to send your submission out for external peer review.

However, before we can send your manuscript to reviewers, we need you to complete your submission by providing the metadata that is required for full assessment. To this end, please login to Editorial Manager where you will find the paper in the 'Submissions Needing Revisions' folder on your homepage. Please click 'Revise Submission' from the Action Links and complete all additional questions in the submission questionnaire.

Once your full submission is complete, your paper will undergo a series of checks in preparation for peer review. After your manuscript has passed the checks it will be sent out for review. To provide the metadata for your submission, please Login to Editorial Manager (https://www.editorialmanager.com/pbiology) within two working days, i.e. by Aug 14 2025 11:59PM.

If your manuscript has been previously peer-reviewed at another journal, PLOS Biology is willing to work with those reviews in order to avoid re-starting the process. Submission of the previous reviews is entirely optional and our ability to use them effectively will depend on the willingness of the previous journal to confirm the content of the reports and share the reviewer identities. Please note that we reserve the right to invite additional reviewers if we consider that additional/independent reviewers are needed, although we aim to avoid this as far as possible. In our experience, working with previous reviews does save time.

If you would like us to consider previous reviewer reports, please edit your cover letter to let us know and include the name of the journal where the work was previously considered and the manuscript ID it was given. In addition, please upload a response to the reviews as a 'Prior Peer Review' file type, which should include the reports in full and a point-by-point reply detailing how you have or plan to address the reviewers' concerns.

During the process of completing your manuscript submission, you will be invited to opt-in to posting your pre-review manuscript as a bioRxiv preprint. Visit http://journals.plos.org/plosbiology/s/preprints for full details. If you consent to posting your current manuscript as a preprint, please upload a single Preprint PDF.

Feel free to email us at plosbiology@plos.org if you have any queries relating to your submission.

Kind regards,

Richard

Richard Hodge, PhD

Senior Editor, PLOS Biology

rhodge@plos.org

PLOS

Empowering researchers to transform science

Carlyle House, Carlyle Road, Cambridge, CB4 3DN, United Kingdom

ORCiD I plosbio.org I @PLOSBiology I Blog

California (U.S.) corporation #C2354500, based in San Francisco

# Peer review: Decision Letter 1

**DOI:** 10.1371/journal.pbio.3003832.r002

**Authors:** Richard Hodge

16 Sep 2025

Dear Dr Palani,

Thank you for your patience while your manuscript "IntAct-U-ExM: Ultrastructure Expansion microscopy of actin networks via an internally-tagged actin" was peer-reviewed at PLOS Biology. Please accept my sincere apologies for the delays that you have experienced during the peer review process. Your study has now been assessed and discussed by the PLOS Biology editors, an Academic Editor with relevant expertise and four independent reviewers. As you will see in their reports at the end of this email, despite noting the interest of the study, the reviewers raise significant concerns about the conclusiveness and conceptual advance of the work.

Based on these reviews, I regret that we will not be able to invite revision of the study, as successfully addressing the concerns raised would entail a significant amount of work (beyond what could be reasonably expected of a revision) and whether the current conclusions would be supported is unclear.

As you will see, although the reviewers agree that the study is well done and that the data are of high quality, they raise several serious and overlapping concerns. Specifically, they raise concerns with the overall strength of the methodological advance given that the IntAct approach has been previously developed. In addition, they note that the manuscript lacks direct and comparative benchmarking to existing actin-labeling methods that are compatible with expansion microscopy to demonstrate a proven advantage. Finally, the reviewers note that the experiments only demonstrate feasibility in well-established organisms and that the manuscript lacks a demonstration of broad applicability across diverse cell types and experimental conditions.

Because producing the required data would require substantial cost and effort with an uncertain outcome, we cannot in good faith invite revision of your study. However, given our and the reviewers' interest in the topic, if further work were to allow you to comprehensively address the reviewer concerns outlined above, we would be willing to consider an extensively revised manuscript as a re-submission. The article would receive a new number and submission date and we would evaluate it in view of any related work published in the interim (applying our 6-month scooping protection policy, of course), but we would make every attempt to engage the same Academic Editor and reviewers in its assessment, and the process would not start from scratch but continue from here.

We appreciate that the scale of the requested additional work is significant and understand that you may prefer to pursue faster publication of this work elsewhere. At this point, your manuscript is no longer under active consideration at PLOS Biology. We might be able to facilitate a faster consideration at another PLOS journal; if this is of interest to you, please let me know.

Please feel free to contact me if you would like to discuss next steps for your manuscript. If you do make the choice to revise and re-submit to PLOS Biology, please provide a point-by-point response to the reviewers' comments when re-submitting. You should include this as a separate file, labelled 'Response to Reviewers'. Please also provide the tracking number of this manuscript [PBIOLOGY-D-25-02426R1] in the 'previous interaction' section of the online submission form and in your cover letter.

I am sorry that we cannot be more positive on this occasion. I thank you for having considered PLOS Biology for publication and hope that you will find the reviewers' feedback helpful as you consider how to proceed with this work.

Best regards,

Richard

Richard Hodge, PhD

Senior Editor, PLOS Biology

rhodge@plos.org

------------------------------------------------------------------------

Reviewer remarks:

Reviewer #1: Expansion microscopy is a revolutionary new sample preparation technique that allows substantially higher resolution of cellular structures. However, since sample preparation includes denaturation of cellular protein, many traditional labeling approaches do not work or require specific chemical modification. This holds true for phalloidin or jasplakiniolide-based labeling of actin structures, which so far has best been achieved by dendrimer-coupled phalloidin that is postfixed into the gel. Here, the authors build on their recent discovery of an internal site in the actin protein that allows for incorporation of a binding epitope while maintaining successful incorporation of actin into filaments. This was an important discovery, as actin-GFP notoriously poorly incorporates into filaments. Here, the authors use actin with an internal alfa-tag for nano body mediated post-expansion labeling of actin structures in S. Pombe, S. Cervisiae and mammalian cells. Importantly, they also show its use for gamma-actin, an understudied actin isoform.

The experiments are well executed and the procedures well documented. The data are of outstanding quality and the quantification convincing. This is certainly work that will be of great use of the community. The level of novelty may not be particularly high, since the actin labeling site was just published last year, but the impact of combining it successfully with ExM is very high and the assay can be repeated easily by anyone, so the usefulness of this study for the field warrants publication in PLoS Biology without delay. The plasmids should be available on add gene.

Reviewer #2: This study introduces an optimized method combining ultrastructural expansion microscopy (U-ExM) with IntAct, an internally tagged actin variant, to visualize actin isoforms at nanoscale resolution in yeast and mammalian cell lines. By expressing ALFA-tagged IntAct and labeling with nanobodies, the authors achieve high-resolution imaging of diverse actin structures, including cortical networks, stress fibers, filopodia, and transient nuclear filaments. The method enables isoform-specific visualization in human osteosarcoma cells and mouse neuroblastoma cells.

The manuscript is clearly articulated, with a logical structure that guides the reader through very informative methods and concise results. The data are of high quality, with clear imaging and robust presentation

- Is there significant methodological innovation?

No. this work builds on prior development of IntAct (PLOS Biol, 2024), that reported its incorporation into native actin filaments and its utility in both fixed and live-cell contexts, now adapted for improved imaging after expansion.

- Does it fill a previously unmet technical or conceptual need?

No. Its adaptation to U-ExM, though competent, does not fully address an unmet technical or conceptual need given the availability of existing actin-labeling tools such as LifeAct, phalloidin derivatives, fluorescent protein fusions, and F-actin chromobodies. Results have not been benchmarked against existing methods

- Is it applicable to a diversity of biological systems with broad impact?

Although demonstrated in yeast and mammalian cell lines, the broader applicability across diverse cell types and experimental conditions such as in the context of genetic or signaling perturbations has not been done.

While technically sound, the study offers a limited incremental advance rather than a transformative methodological innovation.

The potential limitation of the IntAct approach remains that internal tagging or exogenous overexpression may alter actin dynamics, interactions with binding partners, or localization, biasing observed structures.

Reviewer #3: The authors follow up on their highly interesting PLoS Biology paper from last year on the IntAct probes, tools that enable actin labeling with minimal perturbation of function, by demonstrating their compatibility with expansion microscopy. Actin has been notoriously difficult to study at the nanoscale with expansion microscopy due to the lack of probes that survive the expansion process or reliably visualize actin in expanded samples. Thus, this work will be of significant interest to the cytoskeletal community. The manuscript is clearly written, the figures are beautifully presented, and the inclusion of a detailed protocol with reagents and step-by-step instructions will make the approach accessible to many laboratories.

However, there are three concerns that prevent me from recommending publication in PLoS Biology in its current form:

1. The manuscript is presented as a methods paper, but it applies the widely used 4X expansion microscopy technique (U-ExM) to cells expressing IntAct probes, both of which have already been published. As a result, the main new finding is simply that IntAct is compatible with U-ExM. The authors do not demonstrate, in a direct comparison, that IntAct probes are superior to existing methods for visualizing F-actin in expanded samples. Moreover, newer expansion approaches now allow much greater sample expansion (e.g., 10X, 20X). To show advanced feasibility, the authors could test IntAct in one or more of these advanced expansion microscopy applications. Alternatively, the authors could demonstrate that their method as presented provides unique biological insights into actin organization that cannot be obtained otherwise. As it stands, the paper suggests such potential but does not actually advance our understanding of actin biology.

2. A recently preprinted probe, HAK-actin (Mercey et al., bioRxiv 2025), also enables actin visualization in expansion microscopy, though through a different strategy (HAK-actin is an engineered actin-binding small molecule). The authors should directly acknowledge this work and provide a comparison of the strengths and limitations of IntAct versus HAK-actin in expansion microscopy, outlining situations where one may be more advantageous than the other.

3. The IntAct plasmids used in this study remain unavailable through Addgene and can only be obtained by request from the corresponding author, despite that they have been previously published. This limited accessibility will hinder broad adoption of the method, particularly compared with HANK-actin, which is already commercially available. To maximize the impact of this study and encourage use by the community, the IntAct plasmids should be deposited in a public repository such as Addgene.

Reviewer #4: This manuscript describes IntAct-U-ExM, a method that integrates internally tagged actin constructs with U-ExM to enable isoform-specific, high-resolution imaging of actin cytoskeletal networks in yeast and mammalian cells. The technical approach is innovative, and the authors demonstrate applicability to diverse actin structures. However, while the method is technically promising, the manuscript falls short in several key areas, particularly in its quantitative rigor, biological validation, and engagement with the existing literature. The method is currently limited to genetically modified cell lines and transfection efficiency, and the biological consequences of ectopic expression are not adequately addressed. Moreover, despite presenting width measurements of actin bundles, the manuscript does not systematically compare these to established EM data from literature. The work has the potential to publish in PLOS Biology, but further revision and contextualization are necessary to ensure robustness and clarity.

1. Major Points

(1) The manuscript reports actin bundle widths in S. cerevisiae and S. pombe (~74.9 ± 12.2 nm and ~66.2 ± 9.1 nm), but does not adequately compare these values to EM-based measurements. A systematic table or figure comparing the U-ExM data with prior EM findings in similar systems (e.g., yeast actin cables, cortical networks, mammalian stress fibers) would be informative. This omission is surprising given the goal of the method is ultrastructural fidelity. Without this comparison, it is difficult to evaluate the accuracy of the morphology recovered by the technique.

(2) Most imaging experiments rely on plasmid-driven overexpression of IntAct constructs, which can significantly alter actin dynamics, network architecture, and interactions with binding partners. Not to mention transfection efficiency would be much lower than 100%. While one genome-edited knock-in cell line (HT1080) is included, the manuscript lacks any functional validation (e.g., rescue of actin-null phenotype, co-localization with native actin-binding proteins). The authors should discuss more explicitly the well-documented sensitivity of actin systems to dosage perturbation and clearly indicate which results derive from overexpression.

(3) The method is inherently limited to genetically tractable systems. There is no demonstration of use in tissues or primary cells, and no clear path toward compatibility with fixed or clinical samples. While this is a known limitation of genetically encoded tools, it deserves clearer acknowledgment in the Discussion.

(4) Although nanobody-based post-expansion labeling is a logical choice to reduce linkage error, the manuscript does not quantify this advantage. A simple schematic or numerical comparison to conventional antibody labeling (e.g., ~10 nm vs. ~30 nm linkage error post-expansion) would be useful. Resolution claims are supported by FWHM of filament structures, but no Fourier Ring Correlation or equivalent metric is provided. This weakens the case for sub-100 nm resolution. If such analysis is infeasible, the authors should temper the claims.

(5) The manuscript occasionally refers to "isoform specificity," but this is not directly demonstrated (e.g., actin isoform knockouts or comparative staining not shown).

(6) The method combines IntAct with U-ExM, but similar strategies for actin visualization in ExM have emerged recently. For instance, a 2025 study (PMC12021582) reports a phalloidin-based construct for high-resolution ExM imaging of F-actin. Without direct comparisons (e.g., side-by-side tests of labeling efficiency, linear error or resolution against these alternatives), the manuscript risks overstating innovation. A table benchmarking IntAct-U-ExM against these could clarify its unique advantages, such as isoform integration.

2. Minor Points

(1) Some actin structures (e.g., nuclear actin) are shown without much context. Are these consistent with known morphologies or localization patterns? Additional biological discussion would be beneficial.

(2) Figure legends could be more quantitative—e.g., number of cells/filaments analyzed per condition.

# Editor report: Decision Letter 2

**DOI:** 10.1371/journal.pbio.3003832.r003

**Authors:** Richard Hodge

17 Oct 2025

Dear Dr Palani,

Thank you for your patience while my colleagues and I reconsidered our decision regarding your manuscript entitled "IntAct-U-ExM: Ultrastructure Expansion microscopy of actin networks via an internally-tagged actin".

We appreciated the points you raised in the appeal and the additional work that you have already conducted to demonstrate applicability of IntAct-U-ExM in primary mouse hippocampal neurons. Therefore, we would be willing to consider a much-revised version of the manuscript that responds to all of the reviewers' comments. However, after discussions with the editorial team and the academic editor, we would like to make it clear that further consideration would be contingent on providing direct and empirical benchmarking to a few existing actin-labeling methods that are compatible with expansion microscopy to demonstrate a proven advantage (e.g. such as the TRITON probe which is compatible with ExM but only with post-expansion staining).

Given the extent of revision needed, we cannot make a decision about publication until we have seen the revised manuscript and your response to the reviewers' comments. Your revised manuscript is likely to be sent for further evaluation by all or a subset of the reviewers.

In addition to these revisions, you will need to complete some formatting changes, which you will receive in a follow up email. A member of our team will be in touch with a set of requests shortly.

We expect to receive your revised manuscript within 3 months. Please email us (plosbiology@plos.org) if you have any questions or concerns, or would like to request an extension.

At this stage, your manuscript remains formally under active consideration at our journal; please notify us by email if you do not intend to submit a revision so that we may withdraw it.

**IMPORTANT - SUBMITTING YOUR REVISION**

Your revisions should address the specific points made by each reviewer. Please submit the following files along with your revised manuscript:

1. A 'Response to Reviewers' file - this should detail your responses to the editorial requests, present a point-by-point response to all of the reviewers' comments, and indicate the changes made to the manuscript.

*NOTE: In your point-by-point response to the reviewers, please provide the full context of each review. Do not selectively quote paragraphs or sentences to reply to. The entire set of reviewer comments should be present in full and each specific point should be responded to individually, point by point.

You should also cite any additional relevant literature that has been published since the original submission and mention any additional citations in your response.

2. In addition to a clean copy of the manuscript, please also upload a 'track-changes' version of your manuscript that specifies the edits made. This should be uploaded as a "Revised Article with Changes Highlighted" file type.

*Re-submission Checklist*

When you are ready to resubmit your revised manuscript, please refer to this re-submission checklist: https://plos.io/Biology_Checklist

To submit a revised version of your manuscript, please go to https://www.editorialmanager.com/pbiology/ and log in as an Author. Click the link labelled 'Submissions Needing Revision' where you will find your submission record.

Please make sure to read the following important policies and guidelines while preparing your revision:

*Published Peer Review*

Please note while forming your response, if your article is accepted, you may have the opportunity to make the peer review history publicly available. The record will include editor decision letters (with reviews) and your responses to reviewer comments. If eligible, we will contact you to opt in or out. Please see here for more details:

https://blogs.plos.org/plos/2019/05/plos-journals-now-open-for-published-peer-review/

*PLOS Data Policy*

Please note that as a condition of publication PLOS' data policy (http://journals.plos.org/plosbiology/s/data-availability) requires that you make available all data used to draw the conclusions arrived at in your manuscript. If you have not already done so, you must include any data used in your manuscript either in appropriate repositories, within the body of the manuscript, or as supporting information (N.B. this includes any numerical values that were used to generate graphs, histograms etc.). For an example see here: http://www.plosbiology.org/article/info%3Adoi%2F10.1371%2Fjournal.pbio.1001908#s5

*Blot and Gel Data Policy*

We require the original, uncropped and minimally adjusted images supporting all blot and gel results reported in an article's figures or Supporting Information files. We will require these files before a manuscript can be accepted so please prepare them now, if you have not already uploaded them. Please carefully read our guidelines for how to prepare and upload this data: https://journals.plos.org/plosbiology/s/figures#loc-blot-and-gel-reporting-requirements

*Protocols deposition*

To enhance the reproducibility of your results, we recommend that if applicable you deposit your laboratory protocols in protocols.io, where a protocol can be assigned its own identifier (DOI) such that it can be cited independently in the future. Additionally, PLOS ONE offers an option for publishing peer-reviewed Lab Protocol articles, which describe protocols hosted on protocols.io. Read more information on sharing protocols at https://plos.org/protocols?utm_medium=editorial-email&utm_source=authorletters&utm_campaign=protocols

Thank you again for your submission to our journal. We hope that our editorial process has been constructive thus far, and we welcome your feedback at any time. Please don't hesitate to contact us if you have any questions or comments.

Best regards,

Richard

Richard Hodge, PhD

Senior Editor, PLOS Biology

rhodge@plos.org

------------------------------------

Reviewer remarks:

Reviewer #1: Expansion microscopy is a revolutionary new sample preparation technique that allows substantially higher resolution of cellular structures. However, since sample preparation includes denaturation of cellular protein, many traditional labeling approaches do not work or require specific chemical modification. This holds true for phalloidin or jasplakiniolide-based labeling of actin structures, which so far has best been achieved by dendrimer-coupled phalloidin that is postfixed into the gel. Here, the authors build on their recent discovery of an internal site in the actin protein that allows for incorporation of a binding epitope while maintaining successful incorporation of actin into filaments. This was an important discovery, as actin-GFP notoriously poorly incorporates into filaments. Here, the authors use actin with an internal alfa-tag for nano body mediated post-expansion labeling of actin structures in S. Pombe, S. Cervisiae and mammalian cells. Importantly, they also show its use for gamma-actin, an understudied actin isoform.

The experiments are well executed and the procedures well documented. The data are of outstanding quality and the quantification convincing. This is certainly work that will be of great use of the community. The level of novelty may not be particularly high, since the actin labeling site was just published last year, but the impact of combining it successfully with ExM is very high and the assay can be repeated easily by anyone, so the usefulness of this study for the field warrants publication in PLoS Biology without delay. The plasmids should be available on add gene.

Reviewer #2: This study introduces an optimized method combining ultrastructural expansion microscopy (U-ExM) with IntAct, an internally tagged actin variant, to visualize actin isoforms at nanoscale resolution in yeast and mammalian cell lines. By expressing ALFA-tagged IntAct and labeling with nanobodies, the authors achieve high-resolution imaging of diverse actin structures, including cortical networks, stress fibers, filopodia, and transient nuclear filaments. The method enables isoform-specific visualization in human osteosarcoma cells and mouse neuroblastoma cells.

The manuscript is clearly articulated, with a logical structure that guides the reader through very informative methods and concise results. The data are of high quality, with clear imaging and robust presentation

- Is there significant methodological innovation?

No. this work builds on prior development of IntAct (PLOS Biol, 2024), that reported its incorporation into native actin filaments and its utility in both fixed and live-cell contexts, now adapted for improved imaging after expansion.

- Does it fill a previously unmet technical or conceptual need?

No. Its adaptation to U-ExM, though competent, does not fully address an unmet technical or conceptual need given the availability of existing actin-labeling tools such as LifeAct, phalloidin derivatives, fluorescent protein fusions, and F-actin chromobodies. Results have not been benchmarked against existing methods

- Is it applicable to a diversity of biological systems with broad impact?

Although demonstrated in yeast and mammalian cell lines, the broader applicability across diverse cell types and experimental conditions such as in the context of genetic or signaling perturbations has not been done.

While technically sound, the study offers a limited incremental advance rather than a transformative methodological innovation.

The potential limitation of the IntAct approach remains that internal tagging or exogenous overexpression may alter actin dynamics, interactions with binding partners, or localization, biasing observed structures.

Reviewer #3: The authors follow up on their highly interesting PLoS Biology paper from last year on the IntAct probes, tools that enable actin labeling with minimal perturbation of function, by demonstrating their compatibility with expansion microscopy. Actin has been notoriously difficult to study at the nanoscale with expansion microscopy due to the lack of probes that survive the expansion process or reliably visualize actin in expanded samples. Thus, this work will be of significant interest to the cytoskeletal community. The manuscript is clearly written, the figures are beautifully presented, and the inclusion of a detailed protocol with reagents and step-by-step instructions will make the approach accessible to many laboratories.

However, there are three concerns that prevent me from recommending publication in PLoS Biology in its current form:

1. The manuscript is presented as a methods paper, but it applies the widely used 4X expansion microscopy technique (U-ExM) to cells expressing IntAct probes, both of which have already been published. As a result, the main new finding is simply that IntAct is compatible with U-ExM. The authors do not demonstrate, in a direct comparison, that IntAct probes are superior to existing methods for visualizing F-actin in expanded samples. Moreover, newer expansion approaches now allow much greater sample expansion (e.g., 10X, 20X). To show advanced feasibility, the authors could test IntAct in one or more of these advanced expansion microscopy applications. Alternatively, the authors could demonstrate that their method as presented provides unique biological insights into actin organization that cannot be obtained otherwise. As it stands, the paper suggests such potential but does not actually advance our understanding of actin biology.

2. A recently preprinted probe, HAK-actin (Mercey et al., bioRxiv 2025), also enables actin visualization in expansion microscopy, though through a different strategy (HAK-actin is an engineered actin-binding small molecule). The authors should directly acknowledge this work and provide a comparison of the strengths and limitations of IntAct versus HAK-actin in expansion microscopy, outlining situations where one may be more advantageous than the other.

3. The IntAct plasmids used in this study remain unavailable through Addgene and can only be obtained by request from the corresponding author, despite that they have been previously published. This limited accessibility will hinder broad adoption of the method, particularly compared with HANK-actin, which is already commercially available. To maximize the impact of this study and encourage use by the community, the IntAct plasmids should be deposited in a public repository such as Addgene.

Reviewer #4: This manuscript describes IntAct-U-ExM, a method that integrates internally tagged actin constructs with U-ExM to enable isoform-specific, high-resolution imaging of actin cytoskeletal networks in yeast and mammalian cells. The technical approach is innovative, and the authors demonstrate applicability to diverse actin structures. However, while the method is technically promising, the manuscript falls short in several key areas, particularly in its quantitative rigor, biological validation, and engagement with the existing literature. The method is currently limited to genetically modified cell lines and transfection efficiency, and the biological consequences of ectopic expression are not adequately addressed. Moreover, despite presenting width measurements of actin bundles, the manuscript does not systematically compare these to established EM data from literature. The work has the potential to publish in PLOS Biology, but further revision and contextualization are necessary to ensure robustness and clarity.

1. Major Points

(1) The manuscript reports actin bundle widths in S. cerevisiae and S. pombe (~74.9 ± 12.2 nm and ~66.2 ± 9.1 nm), but does not adequately compare these values to EM-based measurements. A systematic table or figure comparing the U-ExM data with prior EM findings in similar systems (e.g., yeast actin cables, cortical networks, mammalian stress fibers) would be informative. This omission is surprising given the goal of the method is ultrastructural fidelity. Without this comparison, it is difficult to evaluate the accuracy of the morphology recovered by the technique.

(2) Most imaging experiments rely on plasmid-driven overexpression of IntAct constructs, which can significantly alter actin dynamics, network architecture, and interactions with binding partners. Not to mention transfection efficiency would be much lower than 100%. While one genome-edited knock-in cell line (HT1080) is included, the manuscript lacks any functional validation (e.g., rescue of actin-null phenotype, co-localization with native actin-binding proteins). The authors should discuss more explicitly the well-documented sensitivity of actin systems to dosage perturbation and clearly indicate which results derive from overexpression.

(3) The method is inherently limited to genetically tractable systems. There is no demonstration of use in tissues or primary cells, and no clear path toward compatibility with fixed or clinical samples. While this is a known limitation of genetically encoded tools, it deserves clearer acknowledgment in the Discussion.

(4) Although nanobody-based post-expansion labeling is a logical choice to reduce linkage error, the manuscript does not quantify this advantage. A simple schematic or numerical comparison to conventional antibody labeling (e.g., ~10 nm vs. ~30 nm linkage error post-expansion) would be useful. Resolution claims are supported by FWHM of filament structures, but no Fourier Ring Correlation or equivalent metric is provided. This weakens the case for sub-100 nm resolution. If such analysis is infeasible, the authors should temper the claims.

(5) The manuscript occasionally refers to "isoform specificity," but this is not directly demonstrated (e.g., actin isoform knockouts or comparative staining not shown).

(6) The method combines IntAct with U-ExM, but similar strategies for actin visualization in ExM have emerged recently. For instance, a 2025 study (PMC12021582) reports a phalloidin-based construct for high-resolution ExM imaging of F-actin. Without direct comparisons (e.g., side-by-side tests of labeling efficiency, linear error or resolution against these alternatives), the manuscript risks overstating innovation. A table benchmarking IntAct-U-ExM against these could clarify its unique advantages, such as isoform integration.

2. Minor Points

(1) Some actin structures (e.g., nuclear actin) are shown without much context. Are these consistent with known morphologies or localization patterns? Additional biological discussion would be beneficial.

(2) Figure legends could be more quantitative—e.g., number of cells/filaments analyzed per condition.

REVIEWS:

# Author comment: Author response to Decision Letter 3

**DOI:** 10.1371/journal.pbio.3003832.r004

31 Mar 2026

Attachment Submitted filename: Response_to_Reviewers_PBIOLOGY-D-25-02426R2.pdf

# Peer review: Decision Letter 3

**DOI:** 10.1371/journal.pbio.3003832.r005

**Authors:** Richard Hodge

6 May 2026

Dear Saravanan,

Thank you for your patience while we considered your revised manuscript "IntAct-U-ExM: Ultrastructure Expansion microscopy of actin networks via an internally-tagged actin" for publication as a Update Article at PLOS Biology. Please accept my apologies for the delays that you have experienced during this round of the peer review process. This revised version of your manuscript has been evaluated by the PLOS Biology editors, the Academic Editor and the original reviewers.

Based on the reviews, we are likely to accept this manuscript for publication, provided you satisfactorily address the remaining points raised by Reviewer #4. In addition, please make sure to address the following data and other policy-related requests that I have provided below (A-E):

(A) We routinely suggest changes to titles to ensure maximum accessibility for a broad, non-specialist readership. In this case, we would suggest a minor edit to the title, as follows. Please ensure you change both the manuscript file and the online submission system, as they need to match for final acceptance:

“IntAct-U-ExM enables super-resolution imaging of isoform-specific actin networks across species”

(B) You may be aware of the PLOS Data Policy, which requires that all data be made available without restriction: http://journals.plos.org/plosbiology/s/data-availability. For more information, please also see this editorial: http://dx.doi.org/10.1371/journal.pbio.1001797

Note that we do not require all raw data. Rather, we ask that all individual quantitative observations that underlie the data summarized in the figures and results of your paper be made available in one of the following forms:

-Supplementary files (e.g., excel). Please ensure that all data files are uploaded as 'Supporting Information' and are invariably referred to (in the manuscript, figure legends, and the Description field when uploading your files) using the following format verbatim: S1 Data, S2 Data, etc. Multiple panels of a single or even several figures can be included as multiple sheets in one excel file that is saved using exactly the following convention: S1_Data.xlsx (using an underscore).

-Deposition in a publicly available repository. Please also provide the accession code or a reviewer link so that we may view your data before publication.

Regardless of the method selected, please ensure that you provide the individual numerical values that underlie the summary data displayed in the following figure panels as they are essential for readers to assess your analysis and to reproduce it:

Figure 1C-D, 3C, 3F, S1C-E, S2A, S2D, S3C

NOTE: the numerical data provided should include all replicates AND the way in which the plotted mean and errors were derived (it should not present only the mean/average values).

(C) Please also ensure that each of the relevant figure legends in your manuscript include information on *WHERE THE UNDERLYING DATA CAN BE FOUND*, and ensure your supplemental data file/s has a legend.

(D) Per journal policy, if you have generated any custom code during the course of this investigation, please make it available without restrictions. Please ensure that the code is sufficiently well documented and reusable, and that your Data Statement in the Editorial Manager submission system accurately describes where your code can be found. More information on our Code Policy, what and how to share can be found here: https://journals.plos.org/plosbiology/s/code-availability

Please note that we cannot accept sole deposition of code in GitHub, as this could be changed after publication. However, you can archive this version of your publicly available GitHub code to Zenodo. Once you do this, it will generate a DOI number, which you will need to provide in the Data Accessibility Statement (you are welcome to also provide the GitHub access information). See the process for doing this here: https://docs.github.com/en/repositories/archiving-a-github-repository/referencing-and-citing-content

(E) Please ensure that your Data Statement in the submission system accurately describes where your data can be found and is in final format, as it will be published as written there.

------------------------------------------------------------------------

As you address these items, please take this last chance to review your reference list to ensure that it is complete and correct. If you have cited papers that have been retracted, please include the rationale for doing so in the manuscript text, or remove these references and replace them with relevant current references. Any changes to the reference list should be mentioned in the cover letter that accompanies your revised manuscript.

In addition to these revisions, you may need to complete some formatting changes, which you will receive in a follow up email. A member of our team will be in touch with a set of requests shortly. If you do not receive a separate email within a few days, please assume that checks have been completed, and no additional changes are required.

We expect to receive your revised manuscript within two weeks.

To submit your revision, please go to https://www.editorialmanager.com/pbiology/ and log in as an Author. Click the link labelled 'Submissions Needing Revision' to find your submission record. Your revised submission must include the following:

- a cover letter that should detail your responses to any editorial requests, if applicable, and whether changes have been made to the reference list

- a Response to Reviewers file that provides a detailed response to the reviewers' comments (if applicable, if not applicable please do not delete your existing 'Response to Reviewers' file.)

- a track-changes file indicating any changes that you have made to the manuscript.

NOTE: If Supporting Information files are included with your article, note that these are not copyedited and will be published as they are submitted. Please ensure that these files are legible and of high quality (at least 300 dpi) in an easily accessible file format. For this reason, please be aware that any references listed in an SI file will not be indexed. For more information, see our Supporting Information guidelines:

https://journals.plos.org/plosbiology/s/supporting-information

*Published Peer Review History*

Please note that you may have the opportunity to make the peer review history publicly available. The record will include editor decision letters (with reviews) and your responses to reviewer comments. If eligible, we will contact you to opt in or out. Please see here for more details:

https://plos.org/published-peer-review-history/

*Press*

Should you, your institution's press office or the journal office choose to press release your paper, please ensure you have opted out of Early Article Posting on the submission form. We ask that you notify us as soon as possible if you or your institution is planning to press release the article.

*Protocols deposition*

To enhance the reproducibility of your results, we recommend that if applicable you deposit your laboratory protocols in protocols.io, where a protocol can be assigned its own identifier (DOI) such that it can be cited independently in the future. Additionally, PLOS ONE offers an option for publishing peer-reviewed Lab Protocol articles, which describe protocols hosted on protocols.io. Read more information on sharing protocols at https://plos.org/protocols?utm_medium=editorial-email&utm_source=authorletters&utm_campaign=protocols

Please do not hesitate to contact me should you have any questions.

Best regards,

Richard

Richard Hodge, PhD

Senior Editor, PLOS Biology

rhodge@plos.org

------------------------------------------------------------------------

Reviewer remarks:

Reviewer #1: The authors addressed my concerns and made an honest effort to address the other reviewers concerns as well. I agree to proceed with publication.

Reviewer #2: The authors have compellingly addressed all the points raised.

Reviewer #3: The manuscript is technically strong, clearly written, and presents a useful extension of existing imaging approaches, but in its current form it still does not quite meet the threshold for PLOS Biology. The central limitation is one of scope: rather than delivering a major biological insight, the study primarily represents a well-executed extension of an already published tool. While it convincingly demonstrates that IntAct can be adapted to U-ExM across multiple systems, the overall contribution reads more as method optimization than as a broad conceptual advance. This impression is reinforced by several points. First, the work builds directly on the prior IntAct study, with the main novelty arising from its combination with U-ExM rather than from the introduction of a fundamentally new principle. Second, although the revised manuscript includes useful additions such as comparisons to HAK-actin and application to primary neurons, much of the data still lacks quantitation (including the comparison to HAK-actin, making it completely subjective) and does not present any clearly novel biological findings that are uniquely enabled by this approach. Overall, the manuscript is solid and publishable, and will likely be valuable to the field, but it appears better suited for an imaging- or methods-focused journal.

Reviewer #4: The revised manuscrip has substantially improved. Residual concerns are minor and can be resolved with text revisions only.

Minor concern:

The median ring spacing of 975 nm for β-IntAct slightly exceeds the expected 5× expansion upper bound of 950 nm, and differs non-trivially from the 866 nm observed for γ-IntAct. The authors should add 1-2 sentences in the Results (around Lines 227-231) explaining whether this isoform difference is expected or reflects measurement scatter, and whether the slight exceedance of the 5× bound is within the variability of local expansion factors.

Each instance in the manuscript where 'isoform-specific' imaging is claimed should be paired with a clear reference to van Zwam et al. (2024) as the source of isoform distribution validation. This ensures readers understand that the specificity claim rests on documented prior work rather than direct demonstration in the current study.

# Author comment: Author response to Decision Letter 4

**DOI:** 10.1371/journal.pbio.3003832.r006

11 May 2026

Attachment Submitted filename: Response_to_Reviewers_PBIOLOGY-D-25-02426R3.pdf

# Editor report: Decision Letter 4

**DOI:** 10.1371/journal.pbio.3003832.r007

**Authors:** Richard Hodge

18 May 2026

Dear Saravanan,

On behalf of my colleagues and the Academic Editor, Carole Parent, I am pleased to say that we can accept your manuscript for publication, provided you address any remaining formatting and reporting issues. These will be detailed in an email you should receive within 2-3 business days from our colleagues in the journal operations team; no action is required from you until then. Please note that we will not be able to formally accept your manuscript and schedule it for publication until you have completed any requested changes.

Please take a minute to log into Editorial Manager at http://www.editorialmanager.com/pbiology/, click the "Update My Information" link at the top of the page, and update your user information to ensure an efficient production process.

PRESS

We frequently collaborate with press offices. If your institution or institutions have a press office, please notify them about your upcoming paper at this point, to enable them to help maximise its impact. If the press office is planning to promote your findings, we would be grateful if they could coordinate with biologypress@plos.org. If you have previously opted in to the early version process, we ask that you notify us immediately of any press plans so that we may opt out on your behalf.

We also ask that you take this opportunity to read our Embargo Policy regarding the discussion, promotion and media coverage of work that is yet to be published by PLOS. As your manuscript is not yet published, it is bound by the conditions of our Embargo Policy. Please be aware that this policy is in place both to ensure that any press coverage of your article is fully substantiated and to provide a direct link between such coverage and the published work. For full details of our Embargo Policy, please visit http://www.plos.org/about/media-inquiries/embargo-policy/.

Thank you again for choosing PLOS Biology for publication and supporting Open Access publishing. We look forward to publishing your study.

Best regards,

Richard

Richard Hodge, PhD

Senior Editor, PLOS Biology

rhodge@plos.org

PLOS

Empowering researchers to transform science

Carlyle House, Carlyle Road, Cambridge, CB4 3DN, United Kingdom

California (U.S.) corporation #C2354500, based in San Francisco
